# Kallikrein‐related peptidase 4 induces cancer‐associated fibroblast features in prostate‐derived stromal cells

**DOI:** 10.1002/1878-0261.12075

**Published:** 2017-08-10

**Authors:** Thomas Kryza, Lakmali M. Silva, Nathalie Bock, Ruth A. Fuhrman‐Luck, Carson R. Stephens, Jin Gao, Hema Samaratunga, Australian Prostate Cancer BioResource, Mitchell G. Lawrence, John D. Hooper, Ying Dong, Gail P. Risbridger, Judith A. Clements

**Affiliations:** ^1^ Australian Prostate Cancer Research Centre – Queensland Translational Research Institute Queensland University of Technology (QUT) Woolloongabba Australia; ^2^ Institute of Health and Biomedical Innovation and School of Biomedical Sciences Queensland University of Technology (QUT) Kelvin Grove Australia; ^3^ Regenerative Dentistry and Oral Biology Oral Health Centre University of Queensland Herston Australia; ^4^ Aquesta Pathology Toowong Australia; ^5^ School of Medicine University of Queensland Herston Australia; ^6^ Australian Prostate Cancer BioResource The Prostate Cancer Research Program Monash University Clayton Australia; ^7^ Prostate Research Group Cancer Program – Biomedicine Discovery Institute Department of Anatomy and Developmental Biology Monash Partners Comprehensive Cancer Consortium Monash University Clayton Australia; ^8^ Cancer Biology and Care Program Translational Research Institute Mater Research Institute – The University of Queensland Woolloongabba Australia; ^9^ Prostate Cancer Translational Research Program Cancer Research Division Peter MacCallum Cancer Centre Parkville Australia; ^10^ Sir Peter MacCallum Department of Oncology The University of Melbourne Parkville Australia

**Keywords:** cancer, cancer‐associated fibroblast, kallikrein‐related peptidase, KLK, prostate cancer, tumour microenvironment

## Abstract

The reciprocal communication between cancer cells and their microenvironment is critical in cancer progression. Although involvement of cancer‐associated fibroblasts (CAF) in cancer progression is long established, the molecular mechanisms leading to differentiation of CAFs from normal fibroblasts are poorly understood. Here, we report that kallikrein‐related peptidase‐4 (KLK4) promotes CAF differentiation. KLK4 is highly expressed in prostate epithelial cells of premalignant (prostatic intraepithelial neoplasia) and malignant lesions compared to normal prostate epithelia, especially at the peristromal interface. KLK4 induced CAF‐like features in the prostate‐derived WPMY1 normal stromal cell line, including increased expression of alpha‐smooth muscle actin, *ESR1* and *SFRP1*. KLK4 activated protease‐activated receptor‐1 in WPMY1 cells increasing expression of several factors (FGF1, TAGLN, LOX, IL8, VEGFA) involved in prostate cancer progression. In addition, KLK4 induced WPMY1 cell proliferation and secretome changes, which in turn stimulated HUVEC cell proliferation that could be blocked by a VEGFA antibody. Importantly, the genes dysregulated by KLK4 treatment of WPMY1 cells were also differentially expressed between patient‐derived CAFs compared to matched nonmalignant fibroblasts and were further increased by KLK4 treatment. Taken together, we propose that epithelial‐derived KLK4 promotes tumour progression by actively promoting CAF differentiation in the prostate stromal microenvironment.

**Abbreviations**
AP: activating peptide
CAF: cancer‐associated fibroblast
CM: conditioned medium
Dkk‐1: Dickkopf‐related protein 1
ECM: extracellular matrix
ELISA: enzyme‐linked immunosorbent assay
ESR1: estrogen receptor‐α
FBS: fetal bovine serum
FGF: fibroblast growth factor
GDF15: growth differentiation factor 15
HGF‐SF: hepatocyte growth factor/scatter factor
HGPIN: high‐grade prostatic intraepithelial neoplasia lesion
IGFBP: insulin‐like growth factor‐binding protein
IGF: insulin‐like growth factor
IHC: immunohistochemistry
IL: interleukin
KLK: kallikrein‐related peptidase
LGPIN: low‐grade prostatic intraepithelial neoplasia lesion
LOX: lysyl oxidase
MMP: matrix metalloproteinase
NPF: normal prostate fibroblast
PAR: protease‐activated receptor
PBS: phosphate‐buffered saline
PCR: polymerase chain reaction
PDGF: platelet‐derived growth factor
PIN: prostatic intraepithelial neoplasia lesion
SFRP1: secreted frizzled‐related protein 1
SMA: smooth muscle actin
TAGLN: transgelin
TGF: transforming growth factor
TME: tumour microenvironment
VEGFA: vascular endothelial growth factor
VIM: vimentin

## 1 Introduction

Malignant tumours are formed from cancer cells in a complex tumour microenvironment (TME) producing a large variety of bioactive factors [growth factors, cytokines and extracellular matrix (ECM) proteins] that regulate tumour growth, angiogenesis and metastasis (Gkretsi *et al*., 2015; Mbeunkui and Johann, 2009; Shiao *et al*., 2016). In solid tumours, stromal cells such as adipocytes, fibroblasts and myofibroblasts are the most predominant nonimmune cell populations composing the TME (Doldi *et al*., 2015; Gandellini *et al*., 2015). In particular, the appearance of cancer‐associated‐fibroblasts (CAFs) is a key step in initiation and progression of tumorigenesis as well as for the development of drug‐resistant capacities in cancer cells (Gandellini *et al*., 2015; Gascard and Tlsty, 2016; Shiao *et al*., 2016). The cellular origin of CAFs is greatly dependent on the tumour type. In the case of prostate cancer (PCa), establishment of reactive stroma is already observable in the premalignant lesion, prostatic intraepithelial neoplasia (PIN), including through differentiation of normal fibroblasts and myofibroblasts surrounding the lesions (Augsten, 2014; Tuxhorn *et al*., 2002). Differentiation of normal cells into CAFs is a complex and dynamic process, which is often summarized as a three‐step process (Madar *et al*., 2013). Firstly, premalignant cells recruit adjacent or distant normal cells through paracrine and endocrine signals; secondly, signals emitted by premalignant cells induce a particular phenotype in normal cells; and finally, persistence signals, produced by premalignant/malignant cells, enable the maintenance, expansion and evolution of CAF populations with cancer progression. In return, CAF populations produce paracrine signals that influence cancer progression (Augsten, 2014; Mbeunkui and Johann, 2009).

Populations of CAFs are heterogeneous between tumours as well as between different compartments and developmental stages of each tumour (Augsten, 2014; De Wever *et al*., 2014; Gascard and Tlsty, 2016; Ishii *et al*., 2011). For instance, it is recognized that CAFs associated with primary tumours are different from those found at metastatic sites (De Wever *et al*., 2014). Although several markers are generally recognized as expressed in CAFs, such as α‐smooth muscle actin (SMA) and fibroblast activation protein α, the heterogeneity of CAFs makes it difficult to determine a specific set of molecular markers to characterize the CAF phenotype. This variability of CAF populations reflects the heterogeneity of signals and associated molecular mechanisms controlling the differentiation of normal cells into CAFs. Several signalling pathways are known to be involved in CAF differentiation, including transforming growth factor‐β (TGF‐β) and interleukin‐6 (IL6). These pathways can induce a partial CAF phenotype in normal prostate fibroblasts (NPFs) *in vitro*, but an ensemble of signals rather than a single factor are necessary to mimic differentiation *in vivo* (Bruzzese *et al*., 2014; Doldi *et al*., 2015; Franco *et al*., 2011; Webber *et al*., 2015). In addition, modification of ECM composition and matrix stiffness during tumour formation induces mechanical signals which, together with soluble factors, stimulate stromal cell activation (De Veirman *et al*., 2014). The identification of other signalling factors required for differentiation of normal cells into CAFs is crucial to understand the processes associated with establishment of the TME.

Proteolytic networks play a central role in establishment of the TME by remodelling the physical environment of cancer cells and regulating their interactions with nonmalignant cells (Mason and Joyce, 2011). The kallikrein‐related peptidases (KLKs) comprise a family of 15 secreted serine proteases involved in a multitude of physiological processes and which are deregulated during cancer progression (Kryza *et al*., 2016; Lawrence *et al*., 2010). In PCa, several KLKs are deregulated, notably KLK3/prostate‐specific antigen, which has been used in PCa diagnosis and tumour recurrence monitoring for over 25 years. In addition, KLK4 is also overexpressed in PCa and involved in processes critical for establishment of the TME and cancer progression (Dong *et al*., 2005; Karakosta *et al*., 2016; Mukai *et al*., 2015; Seiz *et al*., 2010). KLK4 exerts autocrine effects on cancer cells and paracrine effects on surrounding normal cells, in turn regulating key signalling pathways (Gao *et al*., 2007; Mukai *et al*., 2015; Ramsay *et al*., 2008a; Wang *et al*., 2010). Notably, KLK4 activates secreted molecules such as hepatocyte growth factor/scatter factor (HGF‐SF) (Mukai *et al*., 2008, 2015), insulin‐like growth factor (IGF) (Matsumura *et al*., 2005) and TGF‐β (Shahinian *et al*., 2014). KLK4 participates in ECM remodelling (Matsumura *et al*., 2005; Shahinian *et al*., 2014; Zhu *et al*., 2014) and acts directly on target cells through proteolysis of membrane‐tethered proteins, such as the ephrin B4 receptor (Lisle *et al*., 2015) and protease‐activated receptors (PARs) (Gratio *et al*., 2010; Ramsay *et al*., 2008a,b; Wang *et al*., 2010). Interestingly, elevated expression and activation of PARs is associated with progression of several cancers including PCa (Han *et al*., 2011; Ramachandran *et al*., 2012).

In this study, we identified that, in addition to being overexpressed in PCa lesions, KLK4 is also elevated in hyperplastic prostate epithelial cells and PIN lesions, where it can interact with adjacent stromal cells. Through activation of PAR1 expressed in the normal prostate stromal cell line WPMY1, KLK4 regulates the expression of several factors involved in the establishment of the CAF phenotype, stimulates cell proliferation and modulates the secretome of stromal cells, increasing its proangiogenic capacity. We confirmed that these factors are similarly regulated in CAFs compared to matched NPFs and that KLK4 can also regulate this same set of genes in patient‐derived NPFs and CAFs. In view of these results, we propose that the secretion of KLK4 by prostate preneoplastic cells is involved in the induction of the CAF phenotype in prostate normal stromal cells, a key step for the initiation of PCa.

## 2 Materials and methods

### 2.1 Reagents

All reagents and materials were purchased in Australia. PAR‐1 activating peptide (AP1; TFLLR‐NH_2_) and PAR‐2 activating peptide (AP2; SLIGKV‐NH_2_) were purchased from Auspep (Parkville, Vic., Australia). Fura‐2 acetoxymethyl ester was obtained from Thermo Fisher (Newstead, Qld, Australia). Antibodies were purchased from the following vendors: anticytokeratin (high molecular weight; 4βE12; Dako, Campbellfield, Vic., Australia), anti‐TAGLN antibody (HPA019467; Sigma‐Aldrich, Castle Hill, NSW, Australia), anti‐β‐actin antibody (ab8226; Abcam, Melbourne, Vic., Australia), anti‐αSMA (SP171; Sigma‐Aldrich), antivimentin (antiVEM, PA5‐27231; Thermo Fisher), anti‐VEGF (500‐P10‐50; Lonza, Mount Waverley, Vic., Australia), rabbit IgG isotype control and secondary antibodies (Thermo Fisher). DAPI counterstaining compound and CyQuant cell proliferation assay were purchased from Thermo Fisher. The protease inhibitor cocktail and other chemical reagents were purchased from Sigma‐Aldrich, except when specified. The Envision peroxidase system and Fast Red Substrate System were purchased from Dako. All cell culture media and reagents were purchased from Thermo Fisher, Australia, except for fetal bovine serum (FBS), which was from Sigma‐Aldrich.

### 2.2 Tissue/sample preparation and immunohistochemistry (IHC)

**Figure 1:**
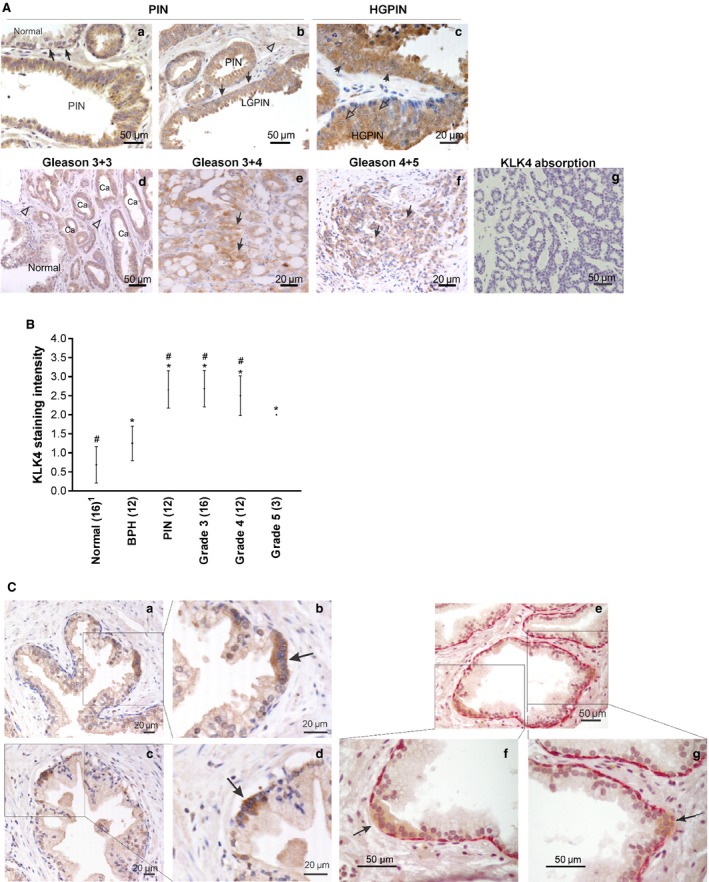
KLK4 expression in the progression of prostate cancer and in foci of atypical suspicious prostate glands. (A) IHC staining of KLK4 in prostate tissues. (a) PIN lesion and adjacent normal glands (Normal, arrows). (b) Low‐grade PIN (LGPIN) with positive staining of basal cells (closed arrows) and no staining in stromal cells (open arrow head). (c) High‐grade PIN (HGPIN) lesions with positive staining in both the nucleus (open arrows) and cytoplasm (arrows) of the secretory cells. (d–f) Strong intensity of KLK4 staining in prostate cancer lesions (Ca or arrows) compared to weak staining of an adjacent normal glands and stroma (Normal and open arrow heads). (g) Anti‐KLK4 antibody absorbance showing no staining, as a negative control. Scale bars are as indicated. (B) Comparison of KLK4 staining intensity in prostate tissues with different Gleason grades. Average staining intensity (●) and SD are shown. Number of region analysed per lesion type is indicated after each lesion name. One‐way ANOVA test, *P < 0.05 compared to normal and ^#^ *P* < 0.05 compared to benign prostatic hyperplasia (BPH). Details can be found in Tables S2 and S3. ^1^Note that the ‘normal’ prostate sample comprises scores for the single normal prostate tissue specimen and 15 tumour‐adjacent normal prostate tissue regions. (C) (a–d) Low and high magnification of cells in adjacent normal gland showing strong KLK4 staining (box), with the high KLK4 foci appearing to have no basal cells (arrow). (e–g) Low and high magnifications of double staining of high molecular weight cytokeratin 34βE12 and KLK4 in normal prostate gland, showing the KLK4 expressing foci (brown) with disappearing basal cells (arrow) compared to the basal cell layer expressing 34βE12 (pink) in the surrounding area. Scale bars are as indicated. PIN, prostatic intraneoplasia; Ca, cancer.

Human prostate tissue samples were obtained as formalin‐fixed and paraffin‐embedded blocks from the archives of the Department of Pathology Royal Brisbane and Women's Hospital, Queensland, Australia. Ethics approval was obtained from the respective Institutional Ethics Committees (QUT1000001171), and informed consent was obtained from all patients. The 32 samples examined by immunohistochemical staining included one normal prostate, 12 benign prostatic hyperplasia (BPH), 19 PCas with different Gleason grades. Five‐micrometre‐thick sections were cut and mounted on 3‐aminopropyltriethoxysilane (Sigma‐Aldrich)‐coated slides. These sections were then subjected to IHC as described previously (Dong *et al*., 2005; Veveris‐Lowe *et al*., 2005) using an affinity‐purified anti‐KLK4 peptide antibody raised against the N terminus (IINGEDCSPHSQ). For better visualization of the basal cells in adjacent normal prostate glands, the Fast Red Substrate System was utilized for the detection of the antibody against high molecular weight cytokeratin 34βE12 as per the company's instructions. Negative controls were performed with mouse or rabbit IgG instead of primary antibodies. Negative controls also included a preincubated anti‐KLK4 antibody with the recombinant KLK4 protein (KLK4/anti‐KLK4, 1/2, w/w, 2 h at room temperature). All sections were examined by a pathologist (H.S.) to confirm histopathological features for comparison of the IHC staining intensity (Fig. 1B, Table S2). The staining intensity of sections was scored according to a scale from 0 to 3 (0, no staining; 1, weak positive; 2, moderately positive; 3, strongly positive) by three independent observers (Loan Bui, Ying Dong and Hemamali Samaratunga). At least five glands or regions in each defined category were examined. One‐way ANOVA and Tukey's multiple comparisons test were used to assess the staining intensity difference among the abovementioned pathological and clinical parameters, with *P* ≤ 0.05 considered to be statistically significant.

### 2.3 Cell lines and primary cells

All cell lines were obtained from ATCC (Manassas, VA, USA): normal prostate stromal cell line (WPMY1), transformed prostate epithelial cell lines (RWPE1 and RWPE2), androgen receptor‐positive, androgen‐responsive PCa cell lines (LNCaP, C42B and 22RV1), androgen receptor‐negative, androgen‐insensitive PCa cell lines (DU145 and PC‐3), human umbilical vein endothelial cells (HUV‐EC‐C) and benign prostate hyperplastic epithelial cells (BPH1). Matched primary NPFs and CAFs were isolated from nonmalignant and tumour regions of patient radical prostatectomy specimens as previously described (Clark *et al*., 2013; Lawrence *et al*., 2013). These samples were obtained with human ethics approval from Monash University (2004/145), Cabrini Hospital (03‐14‐04‐08), under the auspices of the Australian Prostate Cancer BioResource (APCB) and Epworth Hospital (53611). All primary fibroblasts were cultivated in RPMI 1640 containing 5% FBS and 10 ng·mL^−1^ basic fibroblast growth factor (FGF; Merck‐Millipore, Bayswater, Vic., Australia) and used between passage 3 and passage 6 after isolation. All cells were grown in a 5% CO_2_‐humidified atmosphere at 37 °C following the supplier's recommended culture conditions, except when specified.

### 2.4 Recombinant wild‐type KLK4 and mutant KLK4

Full‐length wild‐type recombinant KLK4 was generated as previously described (Ramsay *et al*., 2008a). In addition, the coding sequence of wild‐type KLK4 was modified to produce a double‐mutant KLK4 (mKLK4) corresponding to wild‐type KLK4 with the amino acids serine^207^ and aspartate^116^, in the catalytic triad mutated to an alanine^207^ and asparagine^116^, respectively, in order to inhibit KLK4 proteolytic activity.

### 2.5 Measurement of intracellular Ca^2+^ flux

Cells grown to 80% confluence were washed with phosphate‐buffered saline (PBS), detached nonenzymatically using Versene (Thermo Fisher), resuspended (4 × 10^6^ cells·mL^−1^) and loaded with the fluorescence indicator fura‐2 acetoxymethyl ester (1.0 μm; Thermo Fisher) for 1 h at 37 °C in buffer containing 25 mm HEPES pH 7.4, 121 mm NaCl, 5.4 mm KCl, 0.8 mm MgCl_2_, 1.8 mm CaCl_2_, 5.5 mm glucose, 2.5 mm probenecid and 0.01% (v/v) pluronic acid. Then, cells were washed with PBS and resuspended at 2 × 10^6^ cells·mL^−1^ in the same buffer, lacking fura‐2 and pluronic acid for fluorescence measurements. The ratio of fluorescence at 510 nm after excitation at 340 and 380 nm was monitored using a Polarstar Optima fluorescent plate reader (BMG Labtech Pty Ltd, Mornington, Qld, Australia). Single agonist treatments were performed at 37 °C with KLK4 and mKLK4 (300 nm), AP1 and AP2 (100 μm). Desensitization experiments were performed following the same experimental procedure, and cells were treated successively with two agonists (*t*_1_ = 30 s and *t*_2_ = 480 s). For calcium flux experiments in the presence of PAR1 inhibitor (SHC 79797), cells were pretreated with SHC 79797 (0.3 and 0.7 μm) or vehicle (DMSO) and assays were performed following the same experimental procedure than previously but in the presence of SHC 79797 (same doses than pretreatment). Displayed data are representative of experiments performed in duplicate and repeated on three independent occasions [mean ± standard deviation (SD)].

### 2.6 RNA isolation, reverse transcription and qPCR for gene expression analysis

For the analysis of basal gene expression level in prostate‐derived cell lines, cells were grown until 80% confluent in their respective media and RNA was extracted as described below. For analysis of basal gene expression in NPFs and CAFs, cells were seeded in six‐well plates (50 000 cells per well) in RPMI 1640 containing 5% FBS and 10 ng·mL^−1^ FGF. After 48 h, cells were starved in serum‐free medium overnight and RNA was extracted as described below. To analyse the impact of PAR activation on gene expression in prostate stromal cells (WPMY1, NPF and CAF), cells were seeded in six‐well plates (50 000 cells per well) in their respective medium and grown for 48 h. Then, cells were starved overnight in serum‐free medium before being treated with mKLK4 (20 nm), KLK4 (20 nm), AP1 (100 μm) or AP2 (100 μm) during the specified time. In some experiments, PAR‐1 inhibitor SHC79797 dihydrochloride (0.3 and 0.7 μm; In Vitro Technologies Pty Ltd, Noble Park, Vic., Australia,) was added as an antagonist. For experiments with cells transfected with siRNA, the same protocol was used but siRNA transfection was performed 24 h after seeding (see below).

RNA extraction from cells was performed using the ISOLATE II RNA Kit (Bioline, Eveleigh, NSW, Australia) and reverse‐transcribed (RT) using random hexamer primers and Superscript III (Thermo Fisher). Quantitative PCR (qPCR) was carried out using SYBR Green master mix (Thermo Fisher) and specific primers (Sigma‐Aldrich, Table S1) on a ViiA™ 7 Real‐Time PCR System (Thermo Fisher). mRNA expression was determined using the delta‐delta CT method and using 7SL or RPL32 gene expression as housekeeping genes. Data presented correspond to mean ± standard error (SE) from three independent experiments.

### 2.7 siRNA transfection

In order to knockdown the expression of PAR1 or FGF1, WPMY1 cells were transfected with siRNA targeting PAR‐1 (SMART pool ON‐TARGET plus F2R; Millennium Science, Mulgrave, Qld, Australia), FGF1 (SMART pool ON‐TARGET plus FGF1; Millennium Science) or control‐siRNA (Control pool ON‐TARGET plus cyclophilin B; Millennium Science) following the manufacturer's instructions. Briefly, WPMY1 cells were seeded in six‐well plates (50 000 cells per well) in RPMI 1640 containing 5% FBS. After 24 h, medium was replaced by RPMI 1640 containing 5% FBS, 25 nm of siRNA and 5 μL of DharmaFECT 1 transfection reagent (Millennium Science). After 24 h, medium was replaced with serum‐free RPMI 1640 for PAR agonist treatment or with RPMI 1640 containing 5% FBS for calcium flux assays. Knockdowns were confirmed at the mRNA level by RTqPCR and at the protein level by calcium flux assay for PAR1 and enzyme‐linked immunosorbent assay (ELISA) for FGF1.

### 2.8 ELISAs for FGF1, IL8 and VEGF

The concentration of FGF1 in cellular lysates was measured using a specific FGF1 ELISA (DFA00B; Thermo Fisher). IL8 and VEGF levels in WPMY1‐conditioned medium (CM) were measured using specific IL8 and VEGF ELISAs (900‐M18, 900‐M10; Lonza Australia Pty Ltd, Mt Waverly, Vic., Australia). Briefly, the CM was harvested after treatment, centrifuged to eliminate cellular debris and stored at −80 °C. Cellular proteins were extracted on ice using ELISA lysis buffer (100 mm Tris, pH 7.4, 150 mm NaCl, 1 mm EGTA, 1 mm EDTA, 1% Triton X‐100, 0.5% sodium deoxycholate and protease inhibitor cocktail), and protein concentration was determined by the bicinchoninic acid assay (BCA) (Sigma‐Aldrich). FGF1 ELISA was performed on 30 μg of lysate and ELISAs for IL8 and VEGF were performed on 100 μL of CM using protocols recommended by the manufacturer. Results are expressed in picograms (pg) FGF1 protein/30 μg total protein or in pg IL8 or VEGF·mL^−1^ of CM. The results presented correspond to mean ± SD of three biological replicates.

### 2.9 Western blot

Whole‐cell proteins were extracted on ice using RIPA lysis buffer (150 mm sodium chloride, 1.0% Triton X‐100, 0.5% sodium deoxycholate, 0.1% SDS, 1 mm sodium orthovanadate, 1 mm NaF, 50 mm Tris, pH 8.0, containing protease inhibitor cocktail) and an equal quantity of protein (BCA assay) was separated by SDS/PAGE NuPAGE 4–12% using MOPS buffer (50 mm MOPS, 50 mm Tris base, 0.1% SDS, 1 mm EDTA, pH 7.7) and transferred onto PVDF membranes by liquid transfer. After blocking, PVDF membranes were incubated with respective primary antibodies diluted in TBS‐T (TBS + 0.1% Tween‐20) containing 5% BSA overnight at 4 °C, followed by incubation with species‐appropriate AlexaFluor 680 or IRdye 800‐conjugated secondary antibodies for 45 min. Membranes were scanned on an Odyssey infrared imaging system (LiCor, Mulgrave, Vic., Australia). Consistent protein loading and transfer was determined by reanalysing membranes with either an antiactin or antiVEM antibody, and densitometry analysis was carried out using imagej software (https://imagej.nih.gov/ij/).

### 2.10 Immunofluorescent staining

WPMY1 cells were seeded on poly‐lysine‐coated coverslips and cultured for 48 h in RPMI 1640 containing 5% FBS. Then, cells were starved overnight and treated with mKLK4, KLK4 (20 nm) or AP1 (100 μm) for 48 h. After treatment, cells were fixed and incubated overnight at 4 °C with an anti‐TAGLN or anti‐αSMA antibody, followed by incubation with species‐appropriate secondary antibody coupled with Alexa Fluor^®^ 488 conjugate. Cell nuclei were stained with DAPI before imaging with an Olympus FV1200 laser scanning confocal microscope. For αSMA staining, WPMY1 cells were seeded in 96‐well plates (5000 cells per well) in RPMI 1640 + 5% FBS. After 24 h, cells were treated with mKLK4, KLK4 (20 nm) or AP1 (100 μm) over 6 days (treatment renewed every 48 h). Cells were washed and fixed, incubated as above with an anti‐αSMA antibody and then a goat anti‐mouse IgG (H+L) secondary antibody coupled with Alexa Fluor 488 conjugate and nuclei were stained with DAPI. Imaging was performed with an epifluorescent microscope (IX73 Olympus inverted microscope system) and quantitative measurement was taken using the Incucyte live cell imaging system (Essen BioScience, Ann Arbor, MI, USA). Results presented correspond to mean of fluorescence units ± SD obtained in three independent experiments.

### 2.11 Proliferation of WPMY1 cells

In order to determine the impact of KLK4 and AP1 treatment on stromal cell proliferation, WPMY1 cells were seeded in 96‐well plates (5000 cells per well) in RPMI 1640 + 5% FBS. After 24 h, cells were treated with mKLK4, KLK4 (20 nm) or AP1 (100 μm) for 6 days (treatment renewed every 48 h). After 24, 48, 72 and 96 h of treatment, cells were washed and fixed and nuclei were stained using DAPI. Cells were imaged using the Cytell Cell Imaging System (VWR International Pty Ltd, Tingalpa, Qld, Australia) and the number of cells per well was determined using the cellprofiler software (www.cellprofiler.org) based on DAPI staining. Results presented correspond to mean of number of cells per well ± SD calculated on four biological replicates containing three technical replicates.

### 2.12 Analysis of WPMY1 secretome by cytokine protein array

WPMY1 cells were treated as previously with mKLK4 or KLK4 (20 nm) for 48 h. CM was collected and the WPMY1 cell secretome was analysed using the Proteome Profiler Human XL Cytokine Array Kit (Thermo Fisher) according to the manufacturer's instructions. Results are expressed as mean of relative intensity (%) of duplicate spots, compared to mean intensity of six positive control spots of each array.

### 2.13 Impact of WPMY1 secretome on proliferation of HUV‐EC‐C

WPMY1 cells were cultured as described above and treated with mKLK4, KLK4 (20 nm) or AP1 (100 μm). After 48 h, CM was recovered and spun at 2000 G for 10 min at 4 °C to eliminate cell debris. For live imaging assay, HUV‐EC‐C cells were plated in 96‐well plates (2000 cells) in recommended growth medium. After 24 h, cells were washed with basal medium before being treated with WPMY1 CM. Confluence was followed using Incucyte for 48 h. Results are presented as mean of relative confluence (%) compared to confluence of HUV‐EC‐C cells treated for 24 h with CM from WPMY1 cells treated with mKLK4. Results were calculated for three independent biological replicates. For Cyquant DNA assay, HUV‐EC‐C cells were plated as described above. After 24 h, cells were washed with basal medium before being treated with WPMY1 CM or normal endothelial cell growth medium (EGM) containing isotype IgG control antibody or anti‐VEGF‐neutralizing antibody. The CyQuant proliferation assay was performed after 48 h of treatment. Results are presented as mean of relative fluorescence (%) compared to confluence of HUV‐EC‐C cells treated 48 h with CM of WPMY1 treated with mKLK4 containing isotype IgG control. Results were calculated on three independent biological replicates.

### 2.14 Statistical analysis

Statistical analysis was performed with GraphPad Prism, GraphPad Software, Inc., La Jolla, CA, USA. Unless otherwise stated, statistical analysis was performed using the Kruskal and Wallis test with *P < 0.05, **P < 0.01, ***P < 0.001.

## 3 Results

### 3.1 KLK4 is produced in premalignant and malignant prostatic lesions

KLK4 protein expression in prostate was examined using IHC, with representative images shown in Fig. 1A. Normal glands (normal, Fig. 1Aa) and underlying basal cells (closed arrows, Fig. 1Aa) expressed low levels of KLK4. In prostatic intraepithelial neoplasia (PIN) lesions, KLK4 staining was strong and predominantly localized to the cytoplasm of the secretory cells of prostate glands (Fig. 1Aa–c), and the basal cells of low‐ and high‐grade PIN lesions (LGPIN, HGPIN, closed arrows, Fig. 1Ab–c). KLK4 immunoreactivity was present in the cytoplasm of Gleason grade 3 + 3, 3 + 4, 4 + 5 cancers (Fig. 1Ad–f). Although the major site of KLK4 immunostaining was the cytoplasm, nuclear staining was occasionally detected in the secretory cells of HGPIN lesions (open arrows, Fig. 1Ac). Stromal cells were negative for KLK4 staining (open arrowheads, Fig. 1Ab,d), as were cells from sections preabsorbed with KLK4 peptides prior to immunostaining (Fig. 1Ag), or those treated only with secondary antibody (data not shown). Comparison of KLK4 immunostaining intensity for tissue sections with different histological types is presented in Table S2 and summarized in Fig. 1B. The abundance of KLK4 in BPH, PIN, Gleason 3, 4 and 5 cancers was significantly higher than in the normal prostate (*P *<* *0.05). KLK4 staining in PIN, Gleason 3 and 4 cancers, was higher than in BPH (*P *<* *0.001, one‐way ANOVA; Table S3), whereas KLK4 expression in normal prostate was lower than in BPH (*P *<* *0.023). However, there was no significant difference between KLK4 staining intensity in BPH as compared to Gleason 5 cancer (*P *=* *0.117), or in PIN versus Grade 3 (*P *=* *0.909), Grade 4 (*P *=* *0.667) or Grade 5 (*P* = 0.159) PCas (one‐way ANOVA; Table S3).

KLK4 expression was also analysed in atypical foci (high nuclear/cytoplasmic ratio within cells), suspicious of prostate malignancy, from needle biopsy samples (Fig. 1Ca–g). Interestingly, atypical foci of cells occasionally interspersed within tumour‐adjacent normal glands also showed strong KLK4 immunostaining (closed arrows, Fig. 1Cb,d,f,g), in comparison with the weak or negligible staining in surrounding normal cells. In the 16 adjacent normal prostate regions analysed, six foci of cells displayed strong KLK4 staining within atypical foci; representative sections are shown in Fig. 1Ca–g. Double immunostaining of KLK4 (brown) and high molecular weight cytokeratins, specific for basal cells (red), revealed an absence of the basal cell layer in some instances where strong KLK4 production was observed in luminal atypical foci (closed arrows, Fig. 1Cf–g).

### 3.2 KLK4 specifically activates PAR1 in prostate stromal cells

PAR1 and PAR2 are KLK4 substrates, expressed in both malignant and nonmalignant prostate cells, and involved in PCa progression (Ramsay *et al*., 2008a,b; Wang *et al*., 2010). In order to determine whether KLK4 could regulate stromal cells via activation of PAR1 and/or PAR2, the mRNA expression of these receptors, and that of KLK4, was determined by RTqPCR analysis of a panel of prostate‐derived cell lines (Fig. S2). In agreement with the pattern of KLK4 production observed in prostate biopsy samples (Fig. 1), KLK4 was not expressed in prostate stromal cells (WPMY1) and lowly expressed in normal epithelial cells (RWPE1). However, its expression gradually increased in epithelial cell lines derived from hyperplastic lesions (RWPE2 and BPH1) and in castrate‐sensitive PCa cell lines (LNCaP and 22RV1). In contrast, PAR1 and PAR2 were expressed in epithelial and stromal cells, with the highest relative level of PAR1 in WPMY1 cells.

**Figure 2:**
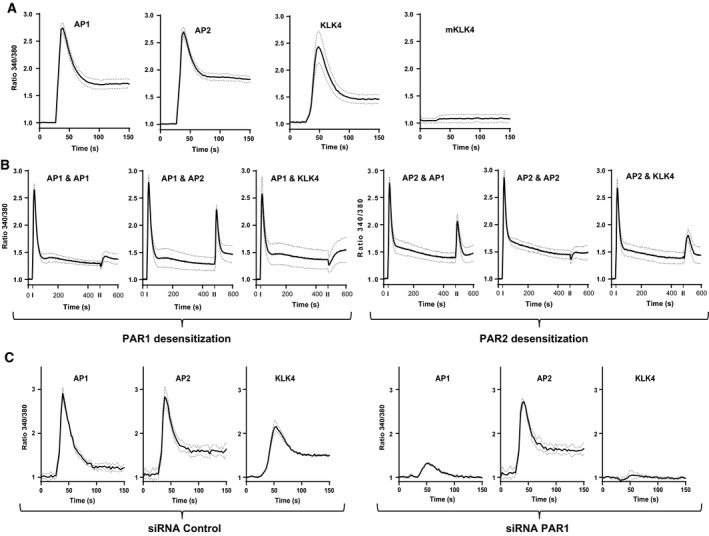
KLK4 can activate PAR1 in prostate‐derived stromal cells. (A) Activation of PARs in WPMY1 cells was analysed by calcium flux assay. Cells were treated with AP1, AP2 (100 μm), KLK4 and mKLK4 (300 nm). Results are expressed as ratio of fluorescence emission between excitation at 340 and 380 nm (ratio 340/380, *y*‐axis) over time (*x*‐axis). (B) Calcium flux assay as in (A). Fluorescence emission was monitored for 600 s and two successive stimulations were made: first stimulation at 30 s with AP1 or AP2 to desensitize PAR1 or PAR2 and second stimulation at 480 s with AP1, AP2 or KLK4. (C) Calcium flux as in A using WPMY1 cells control‐ or PAR1‐siRNA (left and right panel, respectively).

To verify that PAR1 and PAR2 produced by WPMY1 cells were functional, their ability to mobilize intracellular calcium was analysed using agonist peptides specific to PAR1 (AP1) and PAR2 (AP2), recombinant active human KLK4 or a recombinant mutant KLK4 form engineered to be catalytically inactive (mutant KLK4/mKLK4). Both AP1 and AP2 induced an intracellular calcium flux in WPMY1 cells, demonstrating that both PAR1 and PAR2 are functional in this prostate stromal cell line (Fig. 2A). The active form of KLK4, but not mKLK4, also induced a calcium flux in WPMY1 cells (Fig. 2A). This shows that KLK4 activates PAR signalling and that this effect is dependent on its proteolytic activity. To identify the PAR(s) activated by KLK4 on the surface of prostate stromal cells, the same calcium flux assays were used in a desensitization experiment (Holzhausen *et al*., 2006; Kawabata *et al*., 1999). Desensitization of PAR1 suppressed calcium mobilization induced by AP1 or KLK4, but did not affect that induced by AP2 (Fig. 2B). Conversely, AP1 and KLK4 were still able to induce calcium mobilization after PAR2 desensitization, whereas AP2 was not effective (Fig. 2B). This demonstrates that calcium mobilization induced by KLK4 is dependent on PAR1, but not PAR2, activation. To validate this finding, WPMY1 cells were transfected with PAR1‐targeting or control‐siRNA. In WPMY1 control‐siRNA cells, AP1, AP2 and KLK4 induced a calcium flux (Fig. 2C). However, in WPMY1 PAR1‐targeting siRNA cells, only AP2 was able to induce calcium mobilization (Fig. 2C). This confirms that KLK4‐mediated calcium flux in WPMY1 cells is dependent on PAR1 activation, demonstrating that KLK4 can regulate prostate stromal cells through interaction with this receptor. The same conclusion was made from calcium flux assays realized in the presence of a potent selective nonpeptide PAR1 receptor antagonist (SHC79797) (Fig. S1).

### 3.3 KLK4 modulates the expression of FGF1, TAGLN and LOX through activation of PAR1

**Figure 3:**
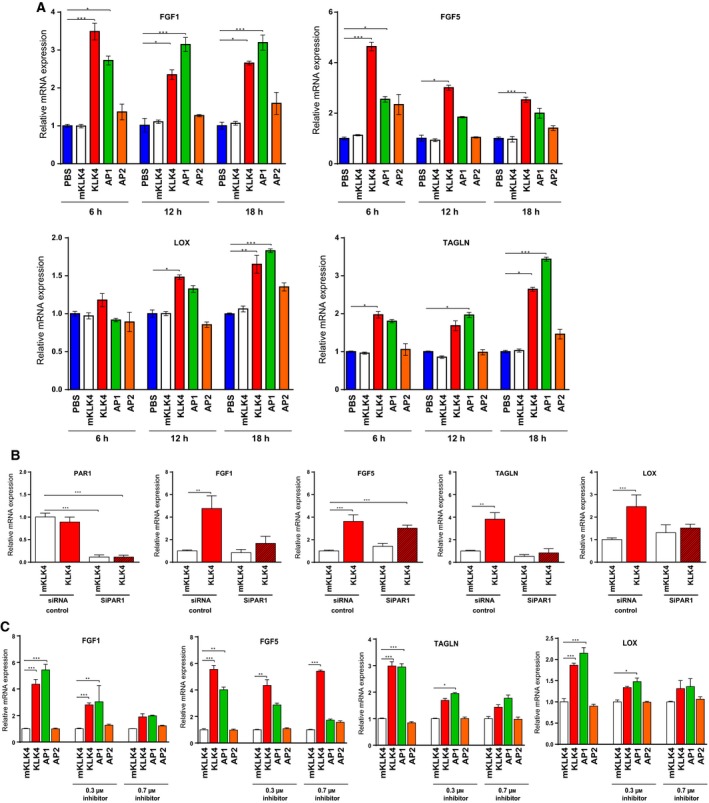
KLK4 regulates gene expression through PAR1 in prostate‐derived stromal cells. (A) Gene expression was studied by RTqPCR in WPMY1 cells treated for 6, 12 or 18 h with mKLK4, KLK4 (20 nm), AP1 or AP2 (100 μm). PBS treatment was used as reference for each time point. Results are presented as mean ± SD of three biological replicates. (B) Gene expression was investigated by RTqPCR in WPMY1 cells transfected with PAR1‐siRNA or control‐siRNA treated with KLK4 or mKLK4 (20 nm) for 18 h. Expression in WPMY1 cells control‐siRNA treated with mKLK4 was used as reference. Results are presented as mean of relative mRNA expression ± SD of three biological replicates. (C) Gene expression was determined by RTqPCR in WPMY1 cells treated for 6 h with mKLK4, KLK4 (20 nm), AP1 or AP2 (100 μm) in the presence of 0.3 and 0.7 μm of PAR1 inhibitor (SHC79797) or vehicle control (DMSO). Gene expression after mKLK4 treatment was used as reference for each concentration of inhibitor. Results are presented as mean ± SD of three biological replicates. *P < 0.05, **P < 0.01, ***P < 0.001 compared to reference.

We have previously identified several genes regulated by KLK4 in WPMY1 cells (R. A. Fuhrman‐Luck & J. A. Clements, unpublished data) and sought to determine whether the deregulation of these genes was mediated by PAR1. The expression of fibroblast growth factor‐1 (FGF1), FGF5, transgelin (TAGLN) and lysyl oxidase (LOX) was significantly up‐regulated by KLK4 or AP1, but not by mKLK4 or AP2 treatment over 18 h (Fig. 3A). Of note, maximum KLK4‐mediated up‐regulation of FGF1 and FGF5 occurred at an earlier time point (6 h) than did up‐regulation of TAGLN and LOX (18 h; Fig. 3A).

To determine the involvement of PAR1 in KLK4‐mediated regulation of the above genes, we analysed the expression of these genes in WPMY1 cells transfected with PAR1‐targeting siRNA, in which a significant reduction in PAR1 mRNA levels (~ 90%) was observed (Fig. 3B). KLK4‐mediated up‐regulation of FGF1, LOX and TAGLN was lower upon PAR1 suppression, as compared to controls, whereas no significant difference in KLK4‐mediated regulation of FGF5 was observed (Fig. 3B). This result suggests that, in WPMY1 cells, KLK4 up‐regulates FGF1, LOX and TAGLN expression through activating PAR1. To confirm this observation, we treated WPMY1 cells with mKLK4, KLK4, AP1 or AP2 in the presence of SHC79797, a potent selective nonpeptide PAR1 receptor antagonist (Fig. 3C). Increasing doses of SHC79797 (0.3 and 0.7 μm) significantly decreased AP1‐mediated up‐regulation of FGF1, FGF5, TAGLN and LOX. However, SHC79797 only inhibited KLK4‐mediated up‐regulation of FGF1, TAGLN and LOX, but not FGF5. This confirms that KLK4 up‐regulates FGF1, TAGLN and LOX expression through PAR1, thereby suggesting that KLK4 might also regulate other signalling pathways independently of PAR1 leading to FGF5 up‐regulation.

### 3.4 KLK4‐mediated activation of PAR1 increases the protein abundance of FGF1 and TAGLN

**Figure 4:**
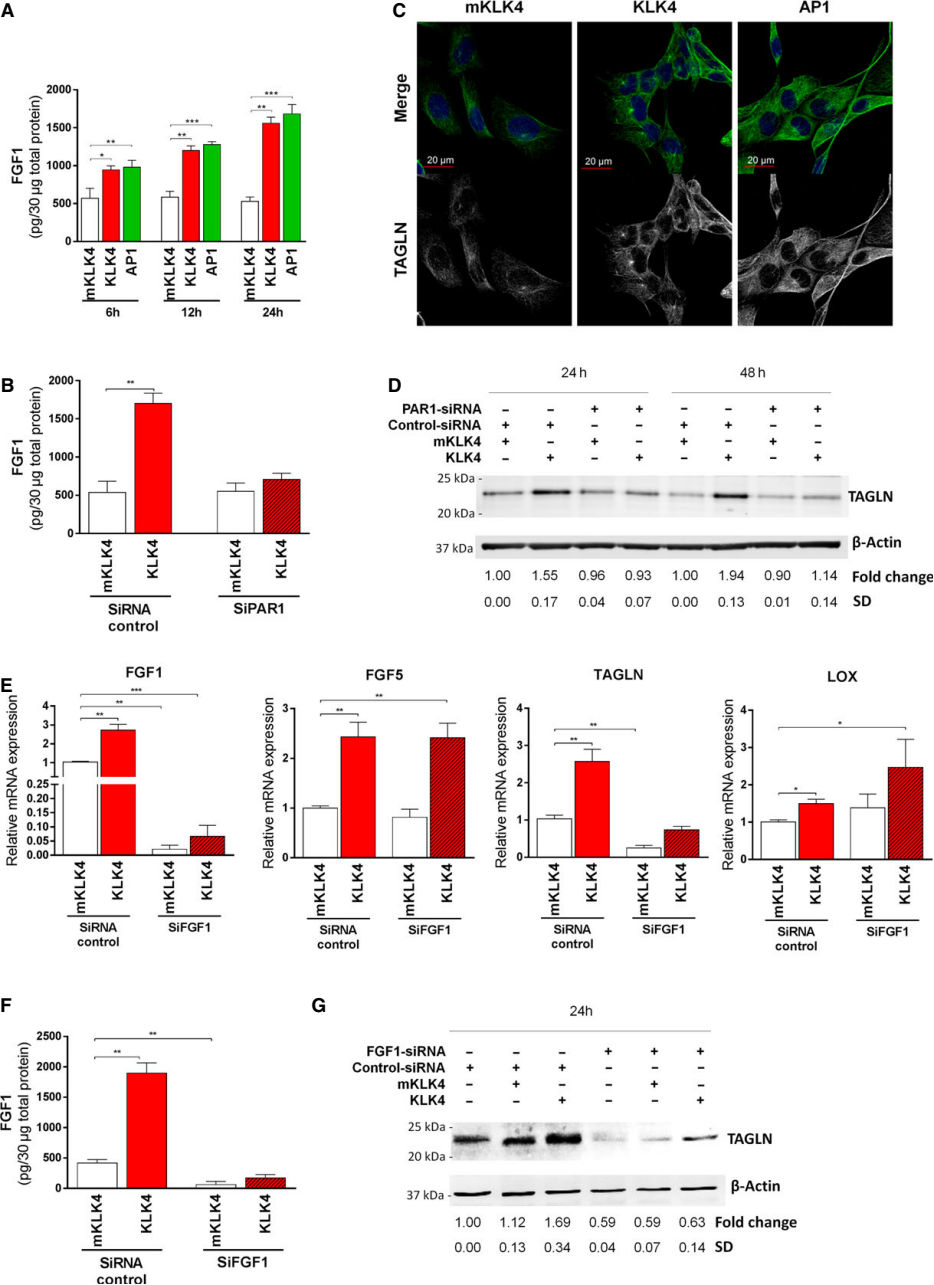
KLK4 regulates protein expression through PAR1 in prostate‐derived stromal cells. (A–B) FGF1 protein in 30 μg of total cellular proteins was determined using FGF1‐ELISA in (A) WPMY1 cells treated for 6, 12 or 18 h with mKLK4, KLK4 (20 nm) or AP1 (100 μm), or (B) WPMY1 cells transfected with PAR1‐siRNA or control‐siRNA and treated with KLK4 or mKLK4 (20 nm) for 24 h. Results are expressed as mean ± SD from three biological replicates. (C) TAGLN expression was determined by immunofluorescent detection in WPMY1 cells treated for 48 h with mKLK4, KLK4 (20 nm) or AP1 (100 μm). Nuclei were stained using DAPI. Representative images are shown, scale bar: 20 μm. (D) TAGLN expression was determined by western blot in WPMY1 cells transfected with PAR1‐siRNA or control‐siRNA and treated for 24 and 48 h with mKLK4 or KLK4 (20 nm). Densitometry analysis was performed using imagej software on three independent experiments. (E–G) WPMY1 cells transfected with FGF1‐siRNA or control‐siRNA were treated for 24 h with mKLK4 or KLK4 (20 nm). (E). Gene expression was obtained by RTqPCR with expression observed for WPMY1 cells control‐siRNA treated with mKLK4 as reference. Results are presented as mean ± SD of three biological replicates. (F) The amount of FGF1 protein in 30 μg of total cellular proteins was determined using FGF1‐ELISA. Results are expressed as mean ± SD calculated on three biological replicates. (G) TAGLN protein expression was determined by western blot as in D. Densitometry analysis was performed using imagej software on three independent experiments. *P < 0.05, **P < 0.01, ***P < 0.001 compared to reference.

The effect of KLK4 on FGF1 and TAGLN production was also analysed at the protein level in cell lysates of WPMY1 cells treated with mKLK4, KLK4 or AP1 over 6, 12 and 24 h. Results showed that both KLK4 and AP1 treatment led to an increase in FGF1 protein levels, compared to treatment with mKLK4 (~ 1.6‐fold at 6 h, ~ twofold at 12 h and ~ threefold at 24 h; Fig. 4A). FGF1 was not detectable in WPMY1 CM (data not shown). FGF1 protein levels in WPMY1 cells transfected with PAR1‐targeting siRNA (or control‐siRNA) after 24‐h treatment with either mKLK4 or KLK4 showed that the KLK4‐mediated increase in FGF1 abundance was completely inhibited in PAR1‐knockdown cells (Fig. 4B).

Analysis of TAGLN protein was first performed by immunofluorescence staining in WPMY1 cells treated with mKLK4, KLK4 or AP1 for 24 h (Fig. 4C). After KLK4 and AP1 treatment, strong specific intracellular staining for TAGLN was observed, showing typical fibrillar organization of this protein (Thompson *et al*., 2012), consistent with its association with the cytoskeleton. Conversely, only weak TAGLN staining was observed in mKLK4‐treated cells. To quantify the differences in TAGLN protein expression, as well as to confirm involvement of PAR1, we used western blot analyses to compare TAGLN abundance in cell lysates from WPMY1 cells transfected with PAR1‐targeting (or control) siRNA, after treatment with either mKLK4 or KLK4 for 24 and 48 h. KLK4 treatment significantly increased the amount of TAGLN in control cells, compared to treatment with mKLK4 (1.55‐fold and 1.94‐fold after 24 and 48 h, respectively; Fig. 4D). However, this effect was completely inhibited upon PAR1 knockdown (Fig. 4D), altogether confirming that KLK4 up‐regulates FGF1 and TAGLN at the protein level, through PAR1 activation.

### 3.5 FGF1 is involved in the regulation of TAGLN expression

FGF1, LOX and TAGLN are downstream effectors of KLK4‐mediated PAR1 activation. Interestingly, KLK4‐mediated up‐regulation of FGF1 occurs before up‐regulation of LOX and TAGLN (Fig. 3A), suggesting that FGF1 may mediate regulation of the latter two proteins. To test this hypothesis, we knocked down FGF1 expression in WPMY1 cells with an FGF1‐targeting siRNA which decreased FGF1 mRNA and protein levels by ~ 90%, as compared to transfection with control‐siRNA (Fig. 4E,F). In these cells, KLK4 was still able to increase FGF1 expression, but to a level significantly lower than in control cells (Fig. 4E,F). Interestingly, knockdown of FGF1 also significantly decreased TAGLN expression as well as its KLK4‐mediated up‐regulation (Fig. 4E,G), whereas the basal expression of FGF5 and LOX genes, as well as their dysregulation by KLK4, was not significantly different than in WPMY1 control‐siRNA cells (Fig. 4E). Specific dysregulation of TAGLN basal expression and KLK4‐mediated up‐regulation of TAGLN after FGF1 knockdown suggest that FGF1 is involved in the regulation of TAGLN expression.

### 3.6 FGF1 and TAGLN are up‐regulated by KLK4 in patient‐derived stromal cells and overexpressed in CAFs compared to NPFs

**Figure 5:**
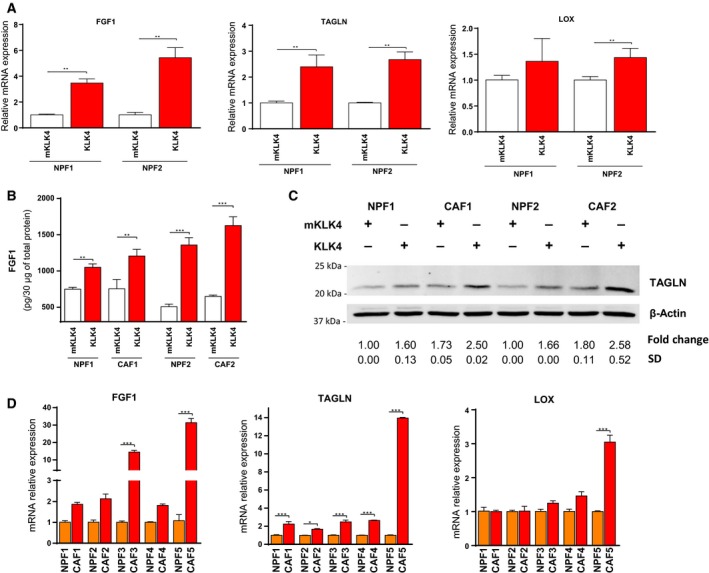
KLK4 regulates gene expression in prostate‐derived NPF/CAF. (A) NPFs isolated from two patients were treated for 24 h with KLK4 and mKLK4 (20 nm) and gene expression analysed (RTqPCR). Gene expression observed for NPF cells treated with mKLK4 was used as reference for each patient. Results are presented as mean ± SD of two biological replicates. (B) The amount of FGF1 protein in 30 μg of total cellular protein in NPF/CAFs from 2 different patients was determined using FGF1‐ELISA. Results are expressed as mean ± SD calculated on three biological replicates. (C) TAGLN protein expression was determined by western blot (as in Fig. 4D). Mann–Whitney test with *P < 0.05, **P < 0.01, ***P < 0.001. (D) Gene expression was analysed in matched NPF/CAF isolated from five patients. Gene expression observed for NPF cells was used as reference for each patient. Results are presented as mean ± SD of three technical replicates. Statistical analysis was performed using one‐way ANOVA test and Bonferroni's multiple comparison, *P < 0.05, **P < 0.01, ***P < 0.001.

To verify that KLK4‐mediated gene regulation was not only limited to WPMY1 cells, we analysed the effect of KLK4 on gene expression in patient‐derived primary cultures of nonmalignant fibroblasts (NPFs) from two patients with PCa (NPF1 and NPF2, Fig. 5A–C). Using the same protocol as for WPMY1 cells, we showed that 24‐h treatment with KLK4 induced a 3.5‐ to 5.0‐fold up‐regulation of FGF1 and a 2.4‐ to 3.0‐fold up‐regulation of TAGLN mRNA expression in NPFs compared to FGF1 and TAGLN expression in NPFs treated with mKLK4 (Fig. 5A). These effects were also observed at the protein level (Fig. 5B,C). KLK4 also significantly increased expression of LOX, but only in NPF2 (Fig. 5A).

To determine whether these genes up‐regulated after KLK4 treatment were associated with a CAF phenotype, we investigated mRNA basal expression of FGF1, LOX and TAGLN in matched pairs of NPFs and CAFs from five patients with PCa (Fig. 5D). The results showed heterogeneity between each NPF/CAF pair analysed. LOX mRNA expression was significantly higher in CAF compared to NPF only in one NPF/CAF pair of five tested. FGF1 was significantly up‐regulated in CAFs in two of three NPF/CAF pairs, while TAGLN was significantly up‐regulated in CAFs in all NPF/CAF pairs tested. Of note, the highest up‐regulation of TAGLN and FGF1 was found in the same NPF/CAF pair (#5).

### 3.7 KLK4 and PAR1 increase expression of CAF markers and cell proliferation in a prostate stromal cell line

To determine whether KLK4 induces a CAF‐like phenotype in prostate stromal cells, we investigated the impact of KLK4 treatment on different CAF‐related features. Compared to NPFs, PCa‐derived CAFs have a higher proliferative rate and a higher expression of different genes which are recognized as markers of CAF phenotype such as αSMA, estrogen receptor‐α (ESR1) and secreted frizzled‐related protein 1 (SFRP1) (Clark *et al*., 2013; Ellem *et al*., 2014; Joesting *et al*., 2005; Ting *et al*., 2015).

**Figure 6:**
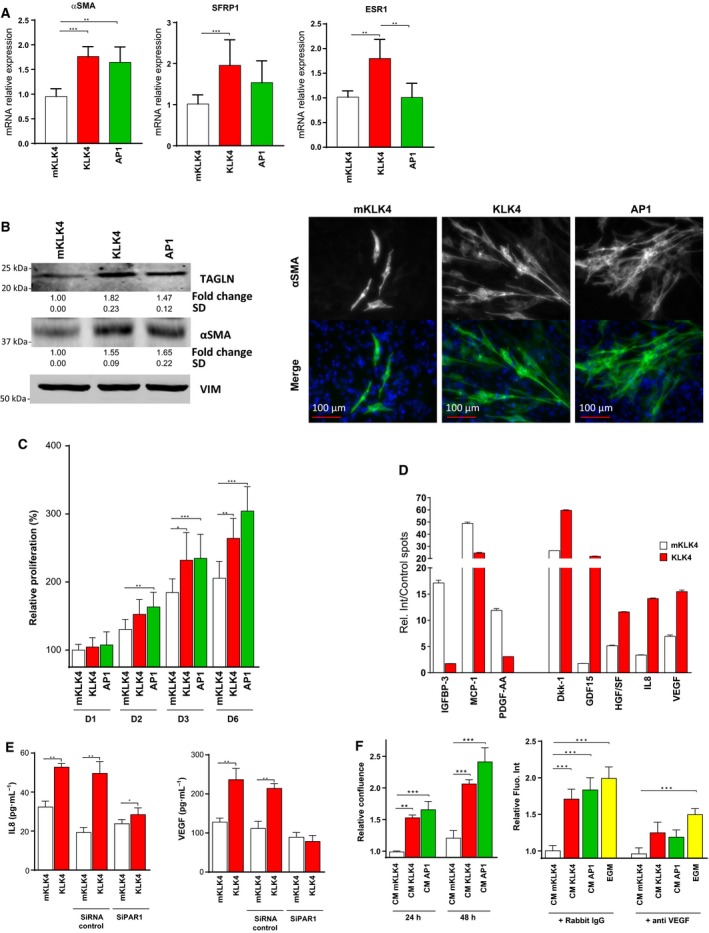
KLK4 induces CAF‐related features in prostate stromal cells through PAR1. (A) Gene expression of αSMA, ESR1 and SFRP1 was determined by RTqPCR in WPMY1 cells treated with mKLK4, KLK4 or AP1 for 24 h. Results are expressed as mean ± SD calculated on three biological replicates, *P < 0.05, **P < 0.01, ***P < 0.001. (B–C) WPMY1 cells were treated every 48 h with mKLK4, KLK4 (20 nm) or AP1 (100 μm) for 6 days. (B) αSMA and TAGLN expression was determined by western blot (left panel). Densitometry analysis was performed using imageJ on three independent experiments. αSMA expression was also determined by immunofluorescent staining (right panel) and the fluorescence quantified (Fig. S2B). (C). Proliferation was measured by direct cell counting using InCell analyzer and cellprofiler software based on nuclei staining (DAPI). Results are presented as mean ± SD of three biological replicates, *P < 0.05, **P < 0.01, ***P < 0.001. (D) Conditioned media (CM) of WPMY1 cells treated for 48 h with KLK4 and mKLK4 (20 nm) were analysed using a protein array (Human XL Cytokine array). For each factor analysed, results are expressed as mean ± SD of relative intensity of duplicate spots compared to mean intensity of six positive control spots present on each array (%). Full analysis of the cytokine array can be found in Table S4. (E) IL8 and VEGF concentrations were determined by ELISA in conditioned media (CM) from wild‐type WPMY1 cells or WPMY1 cells transfected with PAR1‐siRNA or control‐siRNA treated for 24 h with mKLK4 or KLK4 (20 nm). Results are expressed as mean ± SD calculated on three biological replicates. Statistical analysis was performed using *t*‐test, Mann–Whitney with *P < 0.05, **P < 0.01, ***P < 0.001 compared to reference. (F) Effect of CM prepared in E on proliferation of HUVEC cells was analysed. Left panel: HUVEC growth in the presence of WPMY1‐derived CM was followed by live cell imaging (Incucyte) for 48 h. Relative confluence was calculated using confluence at 24 h of HUVEC cells treated with mKLK4‐treated WPMY1's CM as reference. Right panel: HUVEC growth in the presence of WPMY1‐derived CM or endothelial cells growth medium (EGM) was analysed by DNA assay after 48 h of treatment in the presence of an IgG isotype control or a VEGF‐neutralizing IgG. Relative fluorescence intensity was calculated using mKLK4‐treated WPMY1's CM containing IgG isotype control as reference. Results are presented as mean ± SD of three biological replicates, *P < 0.05, **P < 0.01, ***P < 0.001 compared to reference.

Treatment with KLK4 significantly increased mRNA expression of αSMA (1.8‐fold), ESR1 (1.8‐fold) and SFRP1 (1.9‐fold) in WPMY1 cells, compared to cells treated with mKLK4 (or PBS, data not show) (Fig. 6A). Interestingly, AP1 treatment also significantly up‐regulated expression of αSMA (1.6‐fold), suggesting an involvement of this receptor in KLK4‐mediated effect on αSMA expression. Effect of KLK4 and AP1 on αSMA production in WPMY1 cells was confirmed by western blot analysis (1.5‐fold and 1.6‐fold increase, respectively) and immunofluorescence (Fig. 6B, left panel). Similarly, TAGLN production was stimulated by KLK4 and AP1 treatment (1.8‐fold and 1.5‐fold increase, respectively). Expression of VIM, used as loading control, was unchanged. The increase in αSMA abundance after AP1 or KLK4 treatment was also confirmed by immunofluorescence, although only a subpopulation of WPMY1 cells showed high αSMA expression (Fig. 6B, right panel and Fig. S2B).

Secondly, we assessed the effect of KLK4 and AP1 on proliferation of WPMY1 cells. For this purpose, cell density was measured by direct cell counting after 1, 2, 4 and 6 days of treatment with either mKLK4, KLK4 or AP1 (Fig. 6C). Treatment with AP1 significantly increased the proliferation of WPMY1 cells, compared to cells treated with mKLK4, after 2 days of treatment and this persisted after 4 and 6 days. KLK4 also stimulated the proliferation of WPMY1 cells, with a significant pro‐proliferative effect observed after 4 and 6 days.

### 3.8 KLK4 modulates the secretome of WPMY1 prostate stromal cells increasing the secretion of several proangiogenic factors

CAFs regulate tumorigenesis by secreting protumorigenic factors. To determine whether KLK4 modulated the secretome of stromal cells, we analysed the WPMY1 cell secretome, following KLK4 or mKLK4 treatment, using a cytokine array permitting simultaneous analysis of 102 soluble secreted factors. These results identified eight secreted proteins with a relative signal intensity greater than 10% of the mean intensity of control spots, and with a fold difference of −2 ≤ 0 ≥ 2 between KLK4‐ and mKLK4‐treated samples (Fig. 6D). Three cytokines were decreased in CM from cells treated with KLK4, compared to treatment with mKLK4: IGF‐binding protein 3 (IGFBP3; 10‐fold decrease), monocyte chemoattractant protein‐1 (MCP‐1; twofold decrease) and platelet‐derived growth factor‐AA (PDGF‐AA; 3.8‐fold decrease). Conversely, the five other cytokines were increased in CM from WPMY1 cells treated with KLK4, as compared to mKLK4. These were Dkk‐1 (Dickkopf‐related protein 1; 2.2‐fold), GDF15 (growth differentiation factor 15; 12.1‐fold), HGF/SF (2.2‐fold), IL8 (4.2‐fold) and vascular endothelial growth factor (VEGF; 2.2‐fold). Results for other cytokines analysed by the array are presented in Table S4.

To confirm the results obtained with the cytokine array, we determined the levels of IL8 and VEGF in CM from WPMY1 cells using specific ELISAs. In agreement with the cytokine array results, the levels of IL8 were higher in the KLK4‐treated WPMY1 cell secretome, compared to cells treated with mKLK4 (52.8 vs 32.3 pg·mL^−1^; Fig. 6E, left panel). Moreover, although an increase in IL8 was still observed in CM of control cells after KLK4 treatment (49.5 vs 19.2 pg·mL^−1^), PAR1 suppression significantly reduced the KLK4‐mediated increase in IL8 (28.4 vs 23.9 pg·mL^−1^), demonstrating that PAR1 plays a major role in KLK4‐mediated release of IL8 by WPMY1 cells (Fig. 6E, left panel). The same conclusion can be made for KLK4‐mediated VEGF release, as KLK4 treatment of wild‐type and control WPMY1 cells led to an increase in the levels of secreted VEGF (236.5 vs 127.8 pg·mL^−1^ and 214.0 vs 112.5 pg·mL^−1^, respectively), whereas no significant change was observed in WPMY1 cells transfected with PAR1‐targeting siRNA (78.2 vs 89.2 pg·mL^−1^; Fig. 6E, right panel). The effect of KLK4 on IL8 and VEGFA expression was also confirmed at mRNA level by RTqPCR in WPMY1 cells as well as in NPFs/CAFs (Fig. S2C,D).

Interestingly, analysis of mRNA expression levels of these two factors in matched pairs of NPFs and CAFs from five patients with PCa showed that IL8 mRNA expression was very heterogeneous: one patient presented a significantly higher level of IL8 in CAF compared to NPF, whereas two patients presented a significantly lower level in CAF. However, VEGFA mRNA expression level was significantly higher in three NPF/CAF pairs of five tested (Fig. S2E).

### 3.9 KLK4‐treated WPMY1 cells have an increased proangiogenic potential, partially mediated by VEGF

To determine the functional consequences of KLK4‐induced modifications of the WPMY1 secretome in the TME, we analysed the proangiogenic activity of WPMY1 CM via its impact on proliferation of HUVEC endothelial cells. Firstly, live monitoring of endothelial cell growth showed increased proliferation of HUVEC cells treated for 24 and 48 h with CM from KLK4‐ and AP1‐treated WPMY1 cells, compared to cells treated with CM from mKLK4‐treated WPMY1 cells (~ 1.5‐fold at 24 h and ~ twofold at 48 h; Fig. 6F, left panel). Secondly, we analysed HUVEC proliferation after 48‐h treatment with CM from KLK4‐ or AP1‐treated WPMY1 cells, supplemented with a VEGF‐neutralizing antibody or an isotype control (rabbit IgG). Our results confirmed that CM from WPMY1 cells treated with AP1 or KLK4 stimulated proliferation of HUVEC cells, compared to CM from WPMY1 cells treated with mKLK4. In addition, the VEGF‐neutralizing antibody reduced the proliferation of HUVEC cells that was induced by KLK4‐ or AP1‐treated WPMY1 CM, or by normal EGM, as compared to that observed in the presence of the IgG isotype control (Fig. 6F, right panel). This demonstrates that VEGF mediates these proangiogenic effects.

## 4 Discussion

In this report, we demonstrated that KLK4 can induce a CAF‐like phenotype, which is essential for cancer progression, in normal prostate stromal cells, and may be a key contributor to CAF differentiation in PCa. We have shown in normal prostate WPMY1 stromal cells that KLK4 activates PAR1 inducing CAF‐related features such as the modulation of the expression of several factors involved in the establishment of the CAF phenotype, the stimulation of stromal cell growth and the modulation of the stromal cell secretome in favour of a proangiogenic response. These gene expression changes were reproduced in NPFs and matched CAFs from patients with PCa, supporting the biological relevance of our findings. Of note, KLK4 protein is overexpressed in hyperplastic prostate epithelial cells, PIN lesions and malignant epithelium where it could be proteolytically activated by another protease expressed in prostate tissues such as KLK3, KLK11 or a member of MMP family (Bi *et al*., 2010; Yoon *et al*., 2007) and interact with adjacent stromal cells, suggesting that KLK4 secretion from these cells could be a key player in the early stromal differentiation to the CAF phenotype.

Herein, we have confirmed the overexpression of KLK4 in PCa tissues compared to normal prostate as previously demonstrated by several independent studies (Mukai *et al*., 2015; Seiz *et al*., 2010; Veveris‐Lowe *et al*., 2005). In addition, by extending our analysis to nonmalignant prostate lesions, our study is the first to reveal a significant KLK4 overexpression in nonmalignant prostate lesions (BPH, PIN and HGPIN) compared to normal prostate gland. Intriguingly, our analysis also revealed that KLK4 is overexpressed in foci of atypical epithelial cells in normal prostate glands particularly where basal cells are absent. Overproduction of KLK4 by nonmalignant prostate lesions, which are often considered as precursors of PCa, or at least are associated with the presence of PCa (Chrisofos *et al*., 2007; Eminaga *et al*., 2013), and at the interface of epithelial cells and stroma, highlights a possible involvement of KLK4 in mediating interactions between epithelium and stromal cells in the early stages of PCa development.

KLK4 exerts its biological effects through multiple molecular mechanisms, such as regulation of ECM remodelling (Fuhrman‐Luck *et al*., 2016; Zhu *et al*., 2014), control of activity of growth factor‐ and hormone‐related signalling pathways (Mukai *et al*., 2008, 2015; Sanchez *et al*., 2012), modulation of the proteolytic network (Dong *et al*., 2013; Yoon *et al*., 2007) and cleavage of several membrane‐bound proteins (Lisle *et al*., 2015; Matsumura *et al*., 2005; Ramsay *et al*., 2008a,b), notably activation of PARs. Using two different approaches to inhibit PAR1 activation (desensitization and siRNA‐mediated silencing), we identified PAR1 to be predominantly activated by KLK4 in WPMY1 cells. This observation was in total agreement with the higher expression of PAR1 than that of PAR2 in these cells as well as by the higher efficiency of KLK4 to activate PAR1 than PAR2 (Gratio *et al*., 2010; Ramsay *et al*., 2008a). As KLK4 is overproduced by premalignant prostate epithelial cells as well as PCa cells and PAR1 is deregulated in the reactive stroma associated with BPH and PCa (Ramsay *et al*., 2008b; Wang *et al*., 2010; Zhang *et al*., 2009), we hypothesize that KLK4–PAR1 interactions play an important role in prostate stromal cell activation.

KLK4 affects downstream gene expression in WPMY1 cells, regulating expression of several genes (FGF1, FGF5, LOX and TAGLN). Using PAR1 chemical inhibition and siRNA‐mediated PAR1 silencing, we confirmed that KLK4‐mediated up‐regulation of FGF1, LOX and TAGLN was dependent on PAR1, whereas FGF5 was regulated independently of this receptor. Additionally, FGF5 gene was regulated by both the PAR1 agonist and KLK4 although the impact of KLK4 on its expression is PAR1 independent. This leads to the hypothesis that KLK4‐mediated PAR1 activation induces different downstream signalling compared to PAR1‐agonist peptides. Additional experiments must be performed to clearly determine which downstream signalling pathway induced by KLK4‐mediated PAR1 activation is responsible for regulation of FGF1, TAGLN and LOX expression in prostate stromal cells as well as to evaluate the differences between PAR1 activation mediated by activating peptides and KLK4.

Recently, several studies demonstrated that contrary to other members of the FGF family, FGF1 lacks a secretion signal peptide and could exert its effects in an intracrine manner (Bober *et al*., 2016; Delmas *et al*., 2016). As KLK4‐mediated up‐regulation of FGF1 at the mRNA level occurred at an earlier time point compared to LOX and TAGLN up‐regulation, we investigated the involvement of FGF1 in the regulation of LOX and TAGLN expression. Using a FGF1‐siRNA, we showed that this growth factor was necessary for TAGLN expression as well as for KLK4‐mediated TAGLN up‐regulation at both the mRNA and protein levels. In contrast, silencing of FGF1 did not modulate FGF5 and LOX expression as well as the effect of KLK4 on their expression. It would be of interest to further confirm this observed relationship between FGF1 and TAGLN expression and to determine the associated mechanisms.

We have also confirmed that the KLK4 impact on gene expression in prostate stromal cells is not restricted to WPMY1 cells. KLK4 treatment of primary NPF and matched CAF led to up‐regulation of FGF1 and TAGLN gene and protein levels in both NPFs and CAFs from two patients with PCa. However, KLK4‐mediated up‐regulation of IL8 and VEGFA has been confirmed in only one of the two patients tested, demonstrating that heterogeneity of stromal cells will likely modulate the effects of KLK4. Recently, both FGF1 and TAGLN have been identified as highly correlated cancer biomarkers in a cross‐tissue analysis of gene expression in cancer tissues (Kosti *et al*., 2016). We also confirmed that KLK4‐mediated gene regulation is associated with the appearance of the CAF phenotype in prostate fibroblasts, as genes up‐regulated by KLK4 in prostate fibroblasts were also deregulated in prostate‐derived CAFs compared to matching NPFs. Despite the heterogeneity of CAFs, significant TAGLN up‐regulation was observed in five of five CAFs tested compared to NPF. This up‐regulation of TAGLN in CAFs is in agreement with studies demonstrating up‐regulation of this protein in the majority of PCa‐derived CAFs (Webber *et al*., 2016) and in gastric carcinoma‐derived CAFs where it regulates CAF‐mediated metastasis of cancer cells through the regulation of MMP2 expression (Yu *et al*., 2013). TAGLN is an actin‐binding protein involved in the regulation of cell contractibility, which is recognized as an early marker of smooth muscle cells differentiation. However, its role in PCa cells and prostate‐derived stromal cells and CAFs has not been extensively studied (Dvorakova *et al*., 2014). Further studies are required to clearly identify the biological role of TAGLN in the prostate tumour stromal microenvironment, particularly in the promotion of CAF differentiation. Overall, the results obtained with primary NPFs and CAFs confirm that KLK4 can regulate gene expression in prostate‐derived stromal cells notably inducing expression of genes associated with the CAF‐like phenotype.

Investigation of the effect of KLK4 on the prostate stromal cell secretome showed that KLK4 modulates several soluble factors playing important roles in PCa progression. Several secreted factors (IGFBP3, MCP‐1 and PDGF‐AA) showed decreased expression profiles in the secretome of cells treated with KLK4. Interestingly, IGFBP‐3, a protein chaperone of IGFs, and PDGF‐AA, a growth factor involved in the regulation of mesenchymal cell proliferation and tumour progression, are two putative substrates of KLKs in a model of ovarian cancer cells overexpressing KLK4‐7 (Matsumura *et al*., 2005; Prassas *et al*., 2015; Shahinian *et al*., 2014), which may explain their decrease in expression after KLK4 treatment. Conversely, Dkk‐1, GDF15, HGF/SF, IL8 and VEGF levels were increased in the secretome of cells treated with KLK4. HGF/SF, the ligand of HGF receptor MET, is involved in PCa progression stimulating proliferation and migration of cancer cells as well as prostate fibroblasts and CAFs (Han *et al*., 2016; Varkaris *et al*., 2011). Previous studies have demonstrated that KLK4 regulates the activation of the HGF pathway in PCa (Mukai *et al*., 2008, 2015). The increase in HGF/SF release as well as of expression of the HGF receptor MET in WPMY1 cells after KLK4 treatment (data not shown) could explain the increased proliferation of WPMY1 cells after treatment with this protease. IL8 and VEGF are two secreted factors considered as a protumorigenic factor notably because of their role in the regulation of angiogenesis (Culig 2013, Karagiannis *et al*., 2014). Of note, IL8 has been already identified as a regulator of interactions between prostate stromal and epithelial cells in the context of PCa (Kogan‐Sakin *et al*., 2009) and VEGF has been found to be produced at higher levels by CAFs compared to normal fibroblasts (Augsten, 2014; Ishii *et al*., 2011). In our study, we have also shown that both IL8 and VEGF modulations by KLK4 were mediated through a PAR1‐dependent mechanism as previously demonstrated in another fibroblast cell line after treatment with thrombin, the classical activator of PAR1 (Wang *et al*., 2010). Finally, we have also observed that KLK4 or AP1 modulated proangiogenic effect of stromal cell‐conditioned media is dependent, at least partially, on VEGF release. This result reinforces our hypothesis of an induction of a CAF phenotype by KLK4 as CAFs are known to have a high proangiogenic capacity through production and release of proangiogenic molecules (Erez *et al*., 2010; Madar *et al*., 2013; Shiga *et al*., 2015).

**Figure 7:**
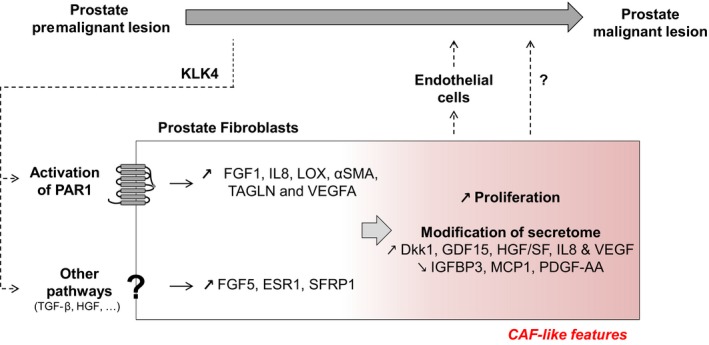
Schematic for the possible involvement of KLK4 in early stages of prostate cancer. KLK4 is produced by premalignant cells in benign prostatic hyperplasia and PIN lesions and acts on prostate fibroblast stromal cells through activation of PAR1 as well as other undetermined pathways conducting to gene regulation of IL8, FGF1, LOX, αSMA, TAGLN and VEGFA (PAR1 dependent) and FGF5, ESR1 and SFRP1 (PAR1 independent). In response to KLK4 stimulation, prostate fibroblast stromal cells present a higher proliferation rate and a modification of their secretome (increase in Dkk‐1, GDF15, HGF/SF and decrease in IGFBP3, MCP1 and PDGF‐AA) in favour of a proangiogenic response (increase in IL8 and VEGF), which could ultimately lead to the development of a proangiogenic microenvironment necessary for prostate cancer progression. We could also hypothesize that modification of prostate fibroblast secretome after KLK4 stimulation could directly influence proliferation/migration/survival of prostate cancer cells.

Overall, our study provides the first evidence for the involvement of KLK4 secreted by premalignant and malignant prostate cells in the induction of CAF‐related features in prostate‐derived stromal cells (Fig. 7). We show that this effect is partially mediated through the activation of the PAR1 receptor expressed in stromal cells adding a new role for this receptor during cancer progression, in addition to its involvement in cancer cell invasion and in the regulation of angiogenesis following its activation by thrombin (Yin *et al*., 2003a,b). Moreover, this study suggests that TAGLN expression could be used as a marker of the CAF phenotype in the context of PCa as its expression is elevated in CAFs compared to NPFs. Future studies will be conducted to define other possible pathways (HGF, TGF‐β) regulated by KLK4 in prostate fibroblasts, to analyse its effect in the presence of other factors regulating the establishment of the CAF phenotype, as well as to confirm its involvement in induction of the CAF phenotype *in vivo*. Finally, evaluation of KLK4 and TAGLN expression in prostate biopsies could determine whether the expression of these two factors in premalignant prostate lesions could predict the development of PCa.

## Author contributions

JAC, JDH, YD and TK conceived and designed the project; TK, LMS, RAFL, CRS and JG acquired the data; TK, JG, HS and YD analysed and interpreted the data; MGL and GPR characterized and provided the primary prostate‐derived fibroblasts and cancer‐associated fibroblasts; TK, NB and JAC wrote the manuscript; all the authors read the manuscript.

## Supporting information


**Fig. S1.** Activation of PARs in WPMY1 cells was analysed by calcium flux assay in presence of 0.3 and 0.7 μm of PAR1 inhibitor (SHC79797) or vehicle control (DMSO).

**Fig. S2.** (A) The expression of KLK4, PAR1 and PAR2 genes have been determined by RTqPCR in different cancerous and noncancerous prostate‐derived cell lines (RWPE1, RWPE2, BPH, LNCaP, 22RV1, PC3, DU145 and WPMY1). Gene expression from PC3 cells were used as reference. Statistical analysis was performed to compare gene expression between WPMY1 and other cell lines tested using One‐way ANOVA test. PAR1 and PAR2 expression levels have been compared in each cell line tested. (B) αSMA protein expression was determined by immunofluorescent staining (Fig. 6B) and the fluorescence quantified using Incucyte analyser. Results are expressed as mean ± SD of relative fluorescent intensity of each field analysed from 3 biological replicates. (C) Gene expression was investigated by RTqPCR in WPMY1 cells transfected with PAR1‐siRNA or control‐siRNA before and after treatment with KLK4 or mKLK4 (20 nm) for 18 h. Expression in WPMY1 cells control‐siRNA treated with mKLK4 was used as reference. Results are presented as mean ± SD of 3 biological replicates. (D) Matched NPF/CAFs isolated from 2 different patients were treated for 24 h with KLK4 and mKLK4 (20 nm). Gene expression was obtained by RTqPCR. Gene expression observed for NPF cells treated with mKLK4 were used as reference for each patient. Results are presented as mean ± SD of 2 biological replicates. Statistical analysis was performed using One‐way ANOVA test Kruskal and Wallis, **P < 0.01, ***P < 0.001 compared to reference. (E) Gene expression was analysed in matched NPF/CAF isolated from 5 different patients in normal culture condition for 48 h. Gene expression observed for NPF cells were used as reference for each patient. Results are presented as mean ± SD of 3 technical replicates. Statistical analysis was performed using One‐way ANOVA test Bonferroni's multiple comparison, *P < 0.05, **P < 0.01, ***P < 0.001 compared to reference.

**Table S1.** RTqPCR primers used.

**Table S2.** Summary of KLK4 expression in different prostate histopathologies. Staining intensity was scored from 0 to 3 (0 for no staining, 1 for weak staining, 2 for moderate, and 3 for strong staining). ^1^Includes only one normal prostate tissue and 15 adjacent normal prostate regions.
**Table S3.** Comparison of KLK4 expression by one‐way ANOVA analysis. ^1^ *P* values are shown for pairwise comparison.

**Table S4.** Summary of relative intensity for each factor, as analysed by cytokine array. For each factor, average intensity was calculated based on two spots present on the array and was divided by the average intensity of positive control spots. The fold change between the secretomes of WPMY1 cells treated with mKLK4 or KLK4 was calculated by dividing corrected mean intensity calculated for each factor.
