# Supplementary figures and images for: Kallikrein‐related peptidase 4 induces cancer‐associated fibroblast features in prostate‐derived stromal cells
Source: Mol Oncol. 2017 Aug 10;11(10):1307–29. doi: 10.1002/1878-0261.12075 (PMC5623815; doi:10.1002/1878-0261.12075)

Supplementary Figure 1

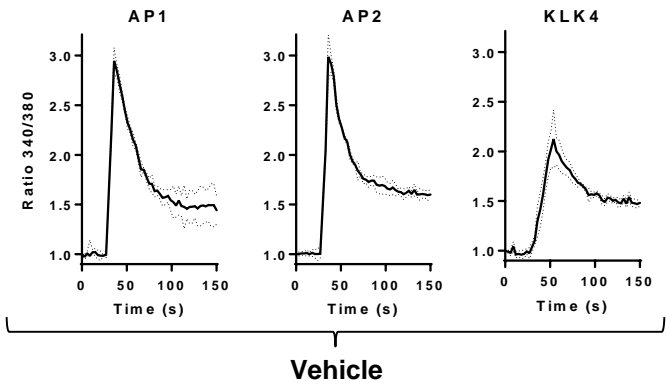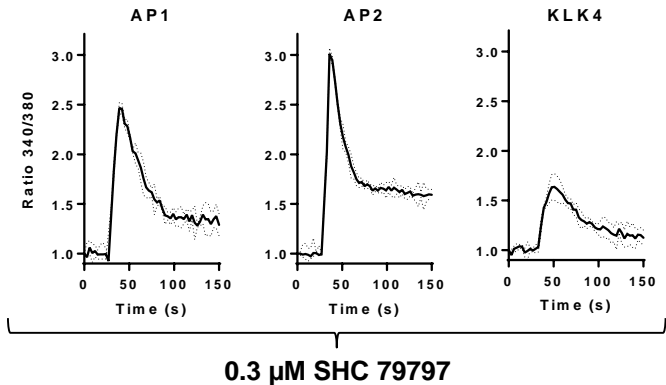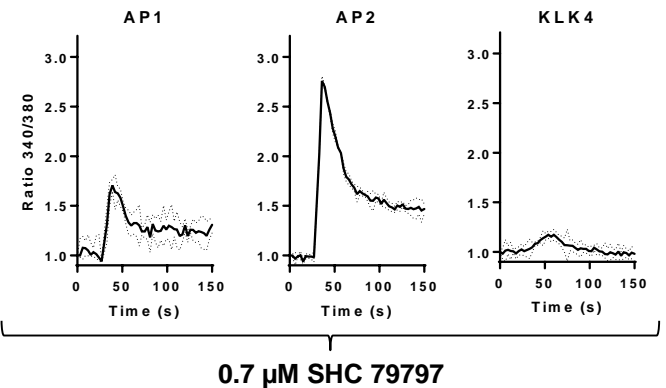

Supplement: Supplementary file 1 — Fig. S1. Activation of PARs in WPMY1 cells was analysed by calcium flux assay in presence of 0.3 and 0.7 μm of PAR1 inhibitor (SHC79797) or vehicle control (DMSO). [file MOL2-11-1307-s001.pdf]

Supplementary Figure 2

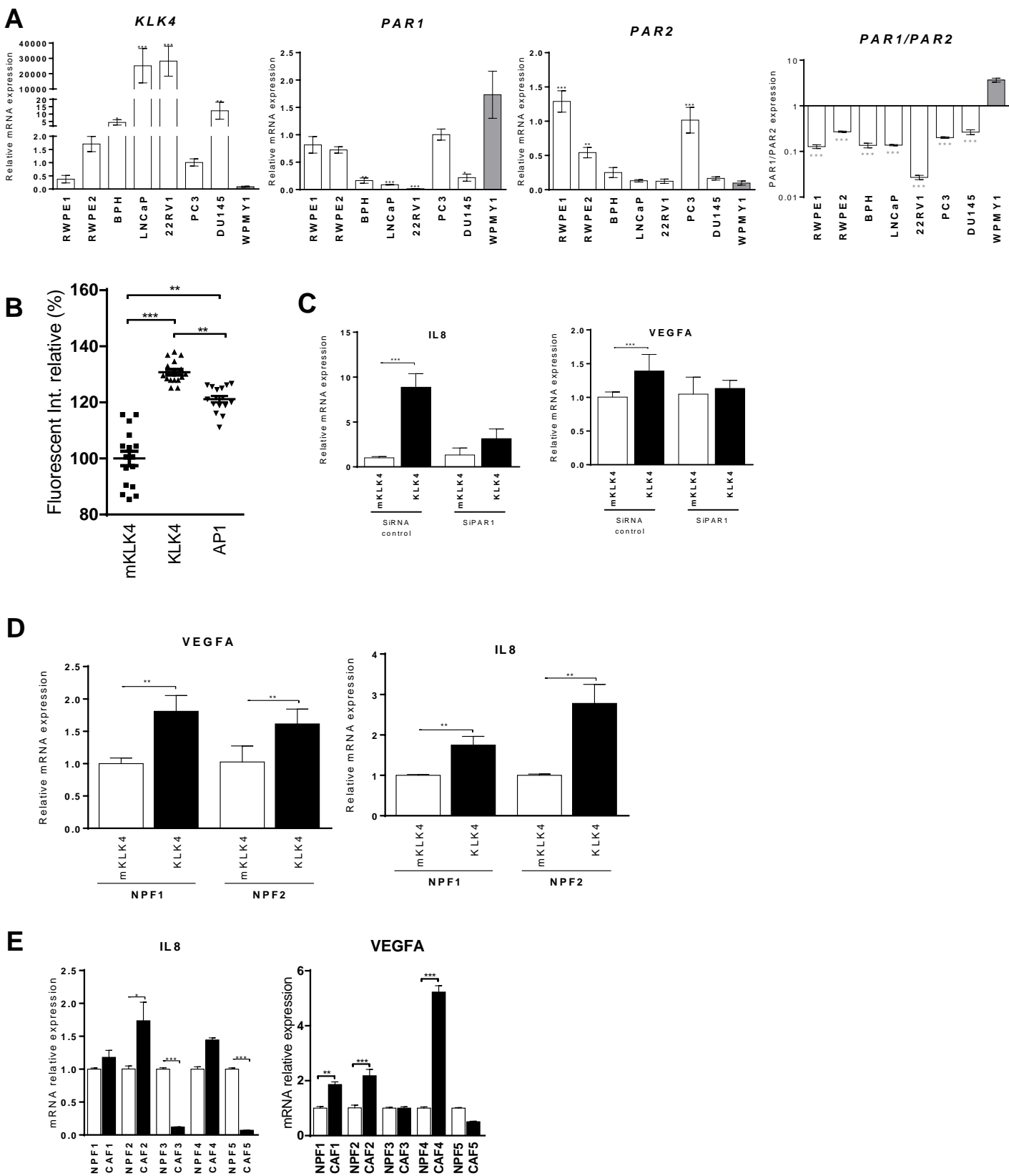

Supplement: Supplementary file 2 — Fig. S2. (A) The expression of KLK4, PAR1 and PAR2 genes have been determined by RTqPCR in different cancerous and noncancerous prostate‐derived cell lines (RWPE1, RWPE2, BPH, LNCaP, 22RV1, PC3, DU145 and WPMY1). Gene expression from PC3 cells were used as reference. Statistical analysis was performed to compare gene expression between WPMY1 and other cell lines tested using One‐way ANOVA test. PAR1 and PAR2 expression levels have been compared in each cell line tested. (B) αSMA protein expression was determined by immunofluorescent staining (Fig. 6B) and the fluorescence quantified using Incucyte analyser. Results are expressed as mean ± SD of relative fluorescent intensity of each field analysed from 3 biological replicates. (C) Gene expression was investigated by RTqPCR in WPMY1 cells transfected with PAR1‐siRNA or control‐siRNA before and after treatment with KLK4 or mKLK4 (20 nm) for 18 h. Expression in WPMY1 cells control‐siRNA treated with mKLK4 was used as reference. Results are presented as mean ± SD of 3 biological replicates. (D) Matched NPF/CAFs isolated from 2 different patients were treated for 24 h with KLK4 and mKLK4 (20 nm). Gene expression was obtained by RTqPCR. Gene expression observed for NPF cells treated with mKLK4 were used as reference for each patient. Results are presented as mean ± SD of 2 biological replicates. Statistical analysis was performed using One‐way ANOVA test Kruskal and Wallis, **P < 0.01, ***P < 0.001 compared to reference. (E) Gene expression was analysed in matched NPF/CAF isolated from 5 different patients in normal culture condition for 48 h. Gene expression observed for NPF cells were used as reference for each patient. Results are presented as mean ± SD of 3 technical replicates. Statistical analysis was performed using One‐way ANOVA test Bonferroni's multiple comparison, *P < 0.05, **P < 0.01, ***P < 0.001 compared to reference. [file MOL2-11-1307-s002.pdf]
