# Supplementary material for: Kallikrein‐related peptidase 4 induces cancer‐associated fibroblast features in prostate‐derived stromal cells
Source: Mol Oncol. 2017 Aug 10;11(10):1307–29. doi: 10.1002/1878-0261.12075 (PMC5623815; doi:10.1002/1878-0261.12075)
Supplement: Supplementary file 3 — Table S1. RTqPCR primers used. [file MOL2-11-1307-s003.pdf]

Supplementary Table 1. Primers used for qPCR analysis

7SL	Forward	5’-ATCGGGTGTCCGCACTAAGTT-3’
	Reverse	5’-CAGCACGGGAGTTTTGACCT-3’
αSMA	Forward	5’-ATGGTGGGAATGGGACAAAA-3’
	Reverse	5’-CGTGAGCAGGGTGGGATG-3’
DKK1	Forward	5’-ATGCGTCACGCTATGTGCT-3'
	Reverse	5’-TTTCCTCAATTTCTCCTCGG-3'
ESR1	Forward	5’-TGATTGGTCTCGTCTGGCGCT-3’
	Reverse	5’-GCACACAAACTCCTCTCCCTGC-3’
FGF1	Forward	5'-AAGCCCGTCGGTGTCCATGG-3'
	Reverse	5'-GATGGCACAGTGGATGGGAC-3'
FGF5	Forward	5'-TTTGCTGTGTCTCAGGGGATT-3'
	Reverse	5'-CTCCCTGAACTTGCAGTCATCT-3'
IL8	Forward	5'-ACTGAGAGTGATTGAGAGTGGAC-3'
	Reverse	5'-AACCCTCTGCACCCAGTTTTC-3'
KLK4	Forward	5'-TGGTAGCTGCAGCCAAATCAT-3'
	Reverse	5'-CTTGGTCGGCCTCAAGACTG-3'
LOX	Forward	5'-TTCTTACCCAGCCGACCAAG-3'
	Reverse	5'-CCTCTGGGTGTTGGCATCAA-3'
PAR1	Forward	5'-TCAGGAGAGAGGGTGAAGCG-3'
	Reverse	5'-CCTGAGAAGAAATGACCGGGG-3'
PAR2	Forward	5'-CTGACTTTCTCTCGGTGCGT-3'
	Reverse	5'-TTCCAGTGACGTGGGATGTG-3'
PDGFA	Forward	5'-CACACCTCCTCGCTGTAGTATTTA-3'
	Reverse	5'-GTTATCGGTGTAAATGTCATCCAA-3'
RPL32	Forward	5’-GCACCAGTCAGACCGATATG-3’
	Reverse	5’-ACTGGGCAGCATGTGCTTTG-3’
SFRP1	Forward	5’-TGGCCCGAGATGCTTAAGTG-3’
	Reverse	5’-ATCCTCAGTGCAAACTCGCT-3’
TAGLN	Forward	5'-GGCTGGTGGAGTGGATCATA-3'
	Reverse	5'-CTTGGAGCCATCAGGGTACA-3'
VEGFA	Forward	5’-CTGAGGAGTCCAACATCACCA-3'
	Reverse	5’-TCATCTCTCCTATGTGCTGGC-3'
