# Supplementary material for: Kallikrein‐related peptidase 4 induces cancer‐associated fibroblast features in prostate‐derived stromal cells
Source: Mol Oncol. 2017 Aug 10;11(10):1307–29. doi: 10.1002/1878-0261.12075 (PMC5623815; doi:10.1002/1878-0261.12075)
Supplement: Supplementary file 4 — Table S2. Summary of KLK4 expression in different prostate histopathologies. Staining intensity was scored from 0 to 3 (0 for no staining, 1 for weak staining, 2 for moderate, and 3 for strong staining). 1Includes only one normal prostate tissue and 15 adjacent normal prostate regions. Table S3. Comparison of KLK4 expression by one‐way ANOVA analysis. 1 P values are shown for pairwise comparison. [file MOL2-11-1307-s004.pdf]

Supplementary Table 2. Summary of KLK4 expression in different prostate histopathologies. Staining intensity was scored from 0 to 3 with (0 for no staining, 1 for weak staining, 2 for moderate and 3 for strong staining.
[1]Includes only one normal prostate tissue and 15 adjacent normal prostate regions.

	Staining Intensity Scores				
Histology	0	1	2	3	Average Score
Normal (16)[1]	5 (27%)	11 (73%)	0	0	0.73
BPH (12)	0	9 (75%)	3 (25%)	0	1.25
PIN (12)	0	0	4 (33%)	8 (67%)	2.67
Grade 3 (16)	0	0	5 (31%)	11 (69%)	2.69
Grade 4 (12)	0	0	6 (50%)	6 (50%)	2.5
Grade 5 (3)	0	0	3 (100%)	0	2

Supplementary Table 3. Comparison of KLK4 expression by One-way ANOVA analysis[1].
[1]*P* Values are shown for pairwise comparison.

	BPH	PIN	Grade 3	Grade 4	Grade 5
PIN	$P < 0.001$	—	—	—	—
Grade 3	$P < 0.001$	$P = 0.909$	—	—	—
Grade 4	$P < 0.001$	$P = 0.667$	$P = 0.667$	—	—
Grade 5	$P = 0.117$	$P = 0.159$	$P = 0.142$	$P = 0.370$	—
Normal	$P = 0.023$	$P < 0.001$	$P < 0.001$	$P < 0.001$	$P = 0.004$
