# Supplementary material for: Kallikrein‐related peptidase 4 induces cancer‐associated fibroblast features in prostate‐derived stromal cells
Source: Mol Oncol. 2017 Aug 10;11(10):1307–29. doi: 10.1002/1878-0261.12075 (PMC5623815; doi:10.1002/1878-0261.12075)
Supplement: Supplementary file 5 — Table S4. Summary of relative intensity for each factor, as analysed by cytokine array. For each factor, average intensity was calculated based on two spots present on the array and was divided by the average intensity of positive control spots. The fold change between the secretomes of WPMY1 cells treated with mKLK4 or KLK4 was calculated by dividing corrected mean intensity calculated for each factor. [file MOL2-11-1307-s005.pdf]

Supplementary Table 4. Summary of relative intensity for each factor analysed by cytokine array. For each factor, average intensity was calculated based on two spots present on the array and was divided by the average intensity of positive control spots. The fold change between the secretomes of WPMY1 cells treated with mKLK4 or KLK4 was calculated by dividing corrected mean intensity calculated for each factor.

Name	mKLK4		KLK4		Fold change
	Mean	SD	Mean	SD	KLK4/mKLK4
Adiponectin	3.92%	1.88%	4.41%	2.46%	1.13
**Aggrecan**	**1.82%**	**0.33%**	**0.70%**	**0.04%**	**0.38**
Angiogenin	8.49%	0.03%	4.50%	0.04%	0.53
**Angiopoietin-1**	**2.67%**	**0.13%**	**1.08%**	**0.06%**	**0.41**
Angiopoietin-2	3.29%	0.05%	1.77%	0.09%	0.54
BAFF	2.51%	0.25%	1.50%	0.28%	0.60
BDNF	3.75%	0.14%	4.46%	0.04%	1.19
C5/C5a	1.69%	0.12%	0.92%	0.09%	0.55
CD14	1.58%	0.01%	0.99%	0.06%	0.63
CD30	3.02%	1.60%	2.28%	0.95%	0.76
CD40L	2.81%	0.48%	3.47%	0.75%	1.24
CHI3L1	1.88%	0.12%	1.22%	0.11%	0.65
CFD	2.36%	0.00%	1.45%	0.14%	0.62
CRP	2.41%	0.02%	1.49%	0.10%	0.62
Cripto-1	2.16%	0.05%	1.17%	0.03%	0.54
Cystatin-C	6.92%	0.46%	6.32%	1.39%	0.91
**Dkk-1**	**26.40%**	**0.02%**	**59.60%**	**0.43%**	**2.26**
DPPIV	1.79%	0.13%	1.57%	0.13%	0.87
EGF	2.75%	0.03%	3.13%	0.11%	1.14
EMMPRIN	17.33%	0.06%	31.72%	0.69%	1.83
**ENA-78**	**3.40%**	**0.05%**	**1.47%**	**0.06%**	**0.43**
Endoglin	4.90%	0.20%	7.30%	0.28%	1.49
Fas Ligand	2.21%	0.02%	1.56%	0.19%	0.71
FGFb	3.19%	0.04%	2.14%	0.02%	0.67
FGF-7	2.01%	0.02%	1.25%	0.06%	0.62
FGF-19	5.35%	0.01%	5.13%	0.36%	0.96
Flt-3 ligand	3.10%	0.19%	3.91%	0.35%	1.26
G-CSF	1.61%	0.05%	1.36%	0.18%	0.84
**GDF-15**	**1.78%**	**0.01%**	**21.71%**	**0.30%**	**12.17**
GM-CSF	2.48%	0.06%	3.25%	0.56%	1.31
GRO-alpha	1.45%	0.12%	0.76%	0.14%	0.53
GH	1.44%	0.10%	0.95%	0.02%	0.66
**HGF-SF**	**5.15%**	**0.12%**	**11.60%**	**0.06%**	**2.25**
ICAM-1	2.10%	0.07%	2.67%	0.26%	1.27
IFN-gamma	3.08%	0.01%	2.65%	0.07%	0.86
IGFBP-2	2.31%	0.19%	2.19%	0.06%	0.95
**IGFBP-3**	**17.11%**	**0.54%**	**1.76%**	**0.01%**	**0.10**
IL-1alpha	3.36%	0.91%	2.66%	0.24%	0.79
IL-1beta	1.65%	0.04%	1.57%	0.01%	0.96
IL-1ra	1.29%	0.03%	1.34%	0.22%	1.04
IL-2	1.51%	0.16%	1.04%	0.14%	0.69
**IL-3**	**1.13%**	**0.18%**	**0.22%**	**0.29%**	**0.20**
IL-4	1.63%	0.14%	1.29%	0.09%	0.79
IL-5	1.54%	0.04%	1.14%	0.02%	0.74
IL-6	2.82%	0.19%	3.37%	0.17%	1.19
**IL-8**	**3.36%**	**0.06%**	**14.15%**	**0.11%**	**4.21**
IL-10	2.45%	0.02%	2.06%	0.05%	0.84
IL-11	3.58%	0.06%	3.65%	0.05%	1.02
IL-12	2.59%	0.08%	1.95%	0.06%	0.75
IL-13	1.62%	0.14%	1.35%	0.11%	0.83
IL-15	1.55%	0.00%	1.55%	0.05%	1.00
IL-16	1.10%	0.01%	0.94%	0.22%	0.85
IL-17	4.41%	0.09%	4.41%	0.28%	1.00

Name	mKLK4		KLK4		Fold change
	Mean	SD	Mean	SD	KLK4/mKLK4
IL-18	1.13%	0.13%	0.42%	0.26%	0.37
IL-19	1.53%	0.07%	1.24%	0.21%	0.81
IL-22	2.15%	0.06%	1.89%	0.11%	0.88
IL-23	1.90%	0.16%	1.78%	0.26%	0.93
IL-24	3.47%	0.12%	3.79%	0.30%	1.09
IL-27	3.05%	0.12%	2.93%	0.10%	0.96
IL-31	2.31%	0.17%	2.10%	0.09%	0.91
IL-32	3.23%	0.05%	3.26%	0.11%	1.01
IL-33	2.12%	0.44%	1.70%	0.19%	0.80
IL-34	1.26%	0.06%	1.17%	0.04%	0.93
IP-10	1.39%	0.11%	1.34%	0.15%	0.96
I-TAC	1.38%	0.08%	0.95%	0.21%	0.69
KLK3	1.52%	0.15%	1.07%	0.26%	0.71
Leptin	1.27%	0.02%	1.25%	0.06%	0.99
LIF	1.45%	0.01%	1.56%	0.00%	1.07
Lipocalin-2	2.87%	0.63%	3.06%	0.19%	1.07
**MCP-1**	**48.76%**	**1.12%**	**24.47%**	**0.50%**	**0.50**
**MCP-3**	**6.04%**	**0.31%**	**2.84%**	**0.16%**	**0.47**
M-CSF	3.08%	0.34%	3.53%	0.05%	1.15
MIF	22.91%	0.22%	22.03%	0.05%	0.96
CXCL9	2.34%	0.41%	2.42%	0.65%	1.03
MIP-1	1.37%	0.17%	1.20%	0.04%	0.88
MIP-3alpha	1.18%	0.01%	1.10%	0.12%	0.94
MIP-3beta	1.09%	0.04%	0.74%	0.15%	0.68
**MMP-9**	**0.90%**	**0.04%**	**0.41%**	**0.06%**	**0.46**
MPO	2.45%	0.18%	2.59%	0.25%	1.06
OPN	3.82%	0.44%	3.12%	0.29%	0.82
**PDGF-AA**	**11.89%**	**0.34%**	**3.08%**	**0.01%**	**0.26**
PDGF-AB	2.38%	0.14%	1.89%	0.15%	0.79
PTX3	34.60%	0.14%	43.44%	0.75%	1.26
CXCL4	3.39%	1.43%	3.45%	1.76%	1.02
RAGE	2.11%	0.17%	2.46%	0.11%	1.16
RANTES	1.63%	0.11%	2.16%	0.02%	1.32
RBP4	2.07%	0.01%	2.03%	0.02%	0.98
Relqxin-2	1.72%	0.04%	2.00%	0.14%	1.16
Resistin	2.51%	0.12%	2.68%	0.23%	1.07
**SDF-1alpha**	**4.68%**	**0.12%**	**1.49%**	**0.20%**	**0.32**
Serpin E1	59.55%	4.26%	75.97%	3.23%	1.28
SHBG	2.57%	0.95%	3.48%	1.23%	1.36
ST2	2.17%	0.12%	1.79%	0.06%	0.82
TARC	2.53%	0.06%	2.36%	0.19%	0.93
TFF3	4.21%	0.24%	5.20%	0.11%	1.23
Tfr	2.96%	0.40%	3.22%	0.63%	1.09
TGF-alpha	1.78%	0.01%	2.04%	0.00%	1.14
TSP-1	2.09%	0.14%	4.00%	0.03%	1.91
TNF-alpha	1.51%	0.02%	1.56%	0.00%	1.03
uPAR	5.62%	0.08%	4.97%	0.07%	0.88
**VEGF**	**6.95%**	**0.24%**	**15.46%**	**0.30%**	**2.22**
Vitamin D	2.83%	0.09%	2.78%	0.04%	0.98
Neg Spot	0.00%	0.14%	0.00%	0.09%	1.00
Ref spot	103.66%	0.26%	94.61%	2.80%	0.91
Ref spot	89.69%	9.45%	91.28%	3.72%	1.02
Ref spot	106.65%	1.84%	114.11%	3.20%	1.07
